# Supplementary material for: Binder driven self-assembly of metal-organic cubes towards functional hydrogels
Source: Nat Commun. 2018 Sep 4;9:3587. doi: 10.1038/s41467-018-05818-w (PMC6123422; doi:10.1038/s41467-018-05818-w)
Supplement: Supplementary file 1 — Supplementary Information [file 41467_2018_5818_MOESM1_ESM.pdf]

Binder driven self-assembly of metal-organic cubes towards functional hydrogels

Sutar et al.

Supplementary Methods

General: 4,5-Imidazoledicarboxylic acid (H_3ImDC), gallium nitrate hexahydrate [$Ga(NO_3)_3.6H_2O$], $TiCl_4$, Zn-dust, 4-hydroxybenzoquinone, 1,2-dibromoethane, N-(2-aminoethyl)-1,3-propanediamine, β-alanine and guanidine hydrochloride were purchased from Sigma-Aldrich chemical Co. Ltd. All the dyes, sulforhodamine G, nile blue,rhodamine B, methylene blue, acridine orange were purchased from Sigma-Aldrich chemical Co. Ltd.Ammonia solution was purchased from SDFCL. All solvents and triethylamine (NEt_3) were obtained from Spectrochem. For UV-Vis experiments, spectroscopic grade solvents were purchased from Spectrochem Pvt. Ltd. (Mumbai, India).

Single-crystal X-ray diffraction: X-ray single-crystal structural data of **1** was collected on a Bruker Smart-CCD diffractometer equipped with a normal focus, 2.4 kW sealed tube X-ray source with graphite monochromated Mo–Kα radiation (λ = 0.71073 Å) operating at 50 kV and 30 mA. The program SAINT[1] was used for integration of diffraction profiles and absorption correction was made with SADABS[2] program. All the structures were solved by SIR 92[3] and refined by full matrix least square method using SHELXL-97.[4]All the non-hydrogen atoms were refined anisotropically and the hydrogen atoms were fixed by HFIX and placed in ideal positions. All calculations were carried out using SHELXL 97, PLATON[5] and WinGX system, Ver 1.70.01.[6]All crystallographic and structure refinement data of **1** are summarized in Supplementary Table 1. Selected bond lengths and angles are displayed in Supplementary Tables 2.

NMR Measurements: ^{1}H NMR is recorded on a Bruker AV-400 spectrometer (400 MHz) with chemical shifts recorded asin parts per million (ppm) and all spectra were calibrated against TMS. Splitting patterns are designated as s, singlet; d, doublet; bs, broad singlet; m, multiplet; t, triplet; dd, doublet of doublet; dt, doublet of triplet.^{13}C NMR is recorded on a Bruker AV-400 spectrometer (100 MHz) with chemical shifts recorded asin parts per million (ppm) and all spectra were calibrated against TMS.

High Resolution Mass Spectrometry (HRMS): High Resolution Mass Spectra (HRMS) were recorded on an Agilent 6538 Ultra High Definition (UHD) Accurate-Mass Q-TOF-LCMS system using electrospray ionization (ESI) technique either in positive mode or negative mode.

Spectroscopic Measurements: UV-Vis spectra were recorded in a Perkin-Elmer lamda 900 spectrometer. Fluorescence studies were accomplished using Perkin Elmer Ls 55 Luminescence spectrometer. Infrared spectral studies were carried out by making samples with KBr pellets using Bruker FT-IR spectrometer.

Powder X-ray Diffraction Measurements: Powder X-ray diffraction (PXRD) were recorded on a Bruker D8 discover instrument using Cu-Kα radiation.

Thermal Stability Measurements: Thermogravimetric analysis (TGA) was carried out using Mettler Toledo TGA 850 instrument under inert atmosphere in the temperature range of 25-800°C at the heating rate of 3°C per min.

Permanent Porosity Measurements: Porosity measurements were carried out using QUANTACHROME QUADRASORD-SI analyser at 77 K for N_2 and 195 K for CO_2.

Morphological Analysis: Morphology studies were carried out using Lica-S440I field emission scanning electron microscopy (FESEM) by placing samples on silicon wafer under vacuum with accelerating voltage of 10 kV. Transmission electron microscopy (TEM) analysis was performed using JEOL JEM-3010 with accelerating voltage of 300 kV. For this analysis the samples were dispersed in ethanol and then drop casted on a carbon coated copper grid.

Energy dispersive X-ray Spectroscopy (EDXS) Analysis: EDXS analysis was performed with an EDAX genesis instrument attached to the FESEM column.

Rheological Measurements: Rheology of hydrogels was performed using a Physicalinterfacial rheology system (IRS).

Binding Affinity Measurements: Binding affinity of different molecular binders to **MOC** was obtained by isothermal titration calorimetric (ITC) studies which were performed in MicroCal iTC200. All solutions were prepared in milli-Q water. All experiments were performed at 25 °C on high-feedback mode with a stirring speed of 750 r.p.m. Long pre-injection-delay (~600 s) were used in order to establish flat baseline.

Zeta Potential Measurements: Zeta potential analysis was carried out in NanoZS (Malvern UK) employing a 532 nm laser.

Synthesis and Characterization

Synthesis of compound S3

In a round bottom flask **S1** (2g, 0.01mol) was dissolved in a 30 ml dry acetone and charged with K_2CO_3 (6 g, 0.04mol) and **S2** (5 ml, 0.07 mol). Reaction mixture was refluxed at 50 °C for 24 hours under inert condition. After completion of reaction, the product was filtered to remove excess K_2CO_3 and filtrate was evaporated. Product was extracted from the aqueous mixture by washing with dichloromethane and dried over anhydrous Na_2SO_4. The pure product was obtained by column chromatography using DCM: hexane (0.4:1) as eluent. Yield: 80 %. ^{1}H NMR (400 MHz, $CDCl_3$, TMS): δ = 7.01-7.09 (m, 6 H), 7.0-7.01 (m, 4 H), 6.91-6.99 (dd, 4H), 6.62-6.67 (dd, 4H), 4.21-4.24 (t, 4 H), 3.57-3.62 (t, 4 H) ppm. HR-MS (ESI):m/z: calculated for $C_{15}H_{13}BrO_2$: 304.0099 $[M]^+$, found: 305.0164 $[M+H]^+$.

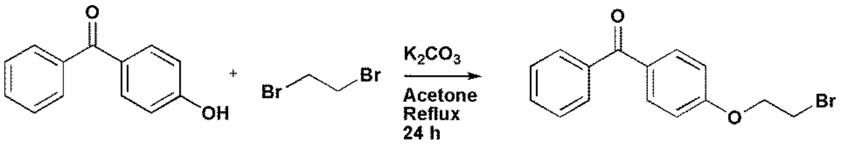

Supplementary Figure 1| Preparation of molecule **S3**.

Synthesis of compound S4.

S3 (1g, 3.27 mmol) was dissolved in 30 ml dry THF and cooled to-78 °C. Zn dust (, 0.411 g, 6.5 mmol) was added to the reaction mixture followed by $TiCl_4$ (0.35 mL, 3.27 mmol) dropwise. Mixture was stirred at -78°C for 1 hour and allowed to room temperature. It is refluxed at 70 °C for 24 hours. Reaction mixture was quenched by adding aqueous K_2CO_3 solution and the product was extracted by DCM. Pure product was obtained by column chromatography using DCM: Hexane (0.5:1) as eluent. Yield: 50 %. ^{1}H NMR (400 MHz, $CDCl_3$, TMS): δ = 7.01-7.09 (m, 6 H), 7.0-7.01 (m, 4 H), 6.91-6.99 (dd, 4H), 6.62-6.67 (dd, 4H), 4.21-4.24 (t, 4 H), 3.57-3.62 (t, 4 H) ppm.^{13}C NMR (100MHz, $CDCl_3$, TMS):δ = 156.49, 144.02, 143.96, 139.73, 137.11, 137.05, 132.59, 131.34, 127.71, 127.59, 126.29, 113.93, 113.81, 67.64, 29.07 ppm. HR-MS (ESI): m/z: calculated for $C_{30}H_{26}Br_2O_2$: 578.0279 $[M]^+$, found: 579.0339 $[M+H]^+$.

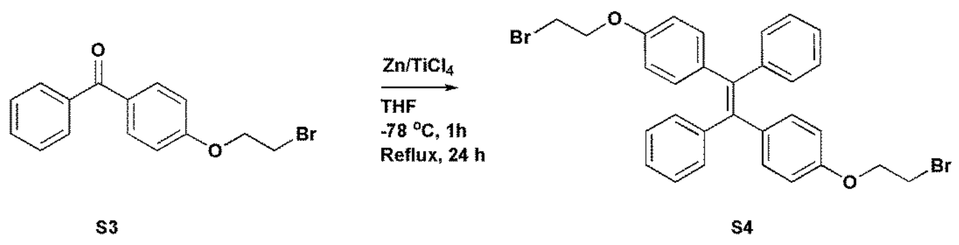

Supplementary Figure 2| Preparation of molecule **S4**.

Synthesis of compound S6.

S4 (100 mg, 0.17 mmol) was dissolved in acetonitrile in a round bottom flask followed by addition of diethylamine (90 μL, 0.86 mmol) and K_2CO_3 (118.8 mg, 0.86 mmol) and the reaction mixture was refluxed for 6 hrs. Excess water is added to the reaction mixture and extracted with ethyl acetate. Organic phase is concentrated under reduced pressure and directly used further. A brown liquid is obtained and it is named **S5**. This brown liquid (**S5**) is dissolved in methanol and stirred with 1.6 N HCl (in methanol) for 15 min. Solvents were evaporated under reduced pressure to obtain pure compound as light brown liquid of DATPE. Yield: 75 %. ^{1}H-NMR (400 MHz, DMSO-d^6, TMS) 10.5 (br, H, NH) 7.1 (m, 4H, CH) 6.8 (m, 3H, CH) 6.7 (m, 2H, CH) 4.2 (m, 2H, OCH_2) 3.4 (d, 2H, CH_2) 3.1 (s, 4H, NCH_2) 1.2 (t, 6H, CH_3). ^{13}C NMR (100MHz, $CDCl_3$, TMS): δ = 155.54, 143.71, 143.58, 139.76, 139.65, 137.56, 132.70, 131.23, 127.75, 127.58, 126.39, 113.80, 113.59, 62.47, 50.51, 47.23, 8.59 ppm. HR-MS (ESI): m/z: calculated for $C_{38}H_{48}N_2O_2$: 564.3716 $[M]^+$, found: 565.3700 $[M+H]^+$.

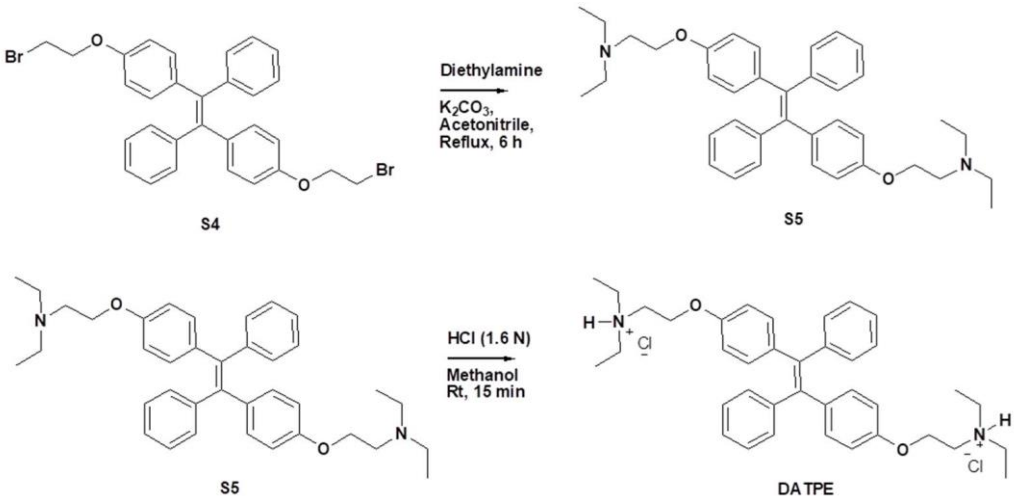

Supplementary Figure 3| Preparation of molecule **S5** andDATPE.

Synthesis of MOC-G1

15 mg **1** is dissolved in 1 ml water. 100 µl aq NH_3 is added into in the solution and sonicated for few minutes. The mixture is kept at room temperature. After 8 hours it forms transparent hydrogel. Formation of gel is confirmed by inversion method. The hydrogel is found to be stable over several months. The xerogel was prepared by air-drying the **MOC-G1** hydrogel. Selected FTIR data of **MOC-G1** xerogel (KBr, cm^{-1}): 3436 (b), 3120 (m), 1663 (s), 1471 (m), 1357 (s), 1102 (s), 844 (m), 658 (m), 492 (m).

Synthesis of MOC-G2

80 µl N-(2-aminoethyl)-1,3-propanediamine (AEPD) was dissolved in 5ml water to make 0.126 M solution of N-(2-aminoethyl)-1,3-propanediamine. 20 mg **1** is dissolved in 500 µl water. 500 µl N-(2-aminoethyl)-1,3-propanediamine solution (0.126 M) was added dropwise and the mixture was sonicated for few minutes. The mixture was kept undisturbed at room temperature. The mixture became viscous after 4-5 hours and formed **MOC-G2** hydrogel after one day. The formation of hydrogel is confirm by inversion test method. The xerogel was prepared by air-drying the **MOC-G2** hydrogel. Selected FTIR data of **MOC-G2** xerogel (KBr, cm^{-1}): 3438 (b), 3110 (m), 1763 (s), 1471 (m), 1355 (s), 1102 (s), 844 (m), 658 (m), 492 (m).

Synthesis of MOC-G3

0.1 M solution of guanidine hydrochloride was prepared by dissolving 24 mg guanidine hydrochloride in 2.5 ml water. 20 mg **1** is dissolved in 300 µl water. 600 µl solution of guanidine hydrochloride was added into the solution of **1** and the mixture was sonicated for few minutes. The mixture was kept undisturbed at room temperature. Transparent hydrogel was formed after 1 day. The formation of hydrogel is confirmed by inversion test method. The xerogel was prepared by air-drying the **MOC-G3** hydrogel. Selected FTIR data of **MOC-G3** xerogel (KBr, cm^{-1}): 3400 (b), 3166 (m), 2781 (m), 1666 (sh), 1473 (sh), 1357 (sh), 1232 (s), 1103 (sh), 1022 (m), 855 (m), 828 (s), 655 (s), 550 (s).

Synthesis of MOC-G4

0.1 M solution of β-alanine was prepared by dissolving 22 mg β-alanine in 2.5 ml water. 20 mg **1** is dissolved in 500 µl water. 500 µl solution of β-alanine was added into the solution of **1** and the mixture was sonicated for few minutes. The mixture was kept undistured at room

temperature. Transparent hydrogel was formed after 1 day. The formation of hydrogel is confirm by inversion test method. The xerogel was prepared by air-drying the **MOC-G4** hydrogel. Selected FTIR data of **MOC-G4** xerogel (KBr, cm^{-1}): 3436 (b), 3220 (m), 1673 (s), 1471 (m), 1367 (s), 1102 (s), 844 (m), 658 (m), 492 (m).

Synthesis of MOC-G5

12 mg **1** was dissolved in 500 µl water. 30 µl DATPE was dissolved in 500 µl water and the solution was drop-wise added into the solution of **1**. The mixture was sonicated for few minutes. An opaque gel was formed instantaneously. The formation of hydrogel is confirm by inversion test method. The xerogel was prepared by air-drying the **MOC-G5** hydrogel. Selected FTIR data of **MOC-G5** xerogel (KBr, cm^{-1}): 3446 (b), 3173 (m), 2992 (m), 2776 (m), 1626 (sh),n 1475 (sh), 1337 (s), 1240 (s), 1106 (sh), 860 (m), 654 (m).

Synthesis of MOC-GM1, MOC-GM2, MOC-GM3, and MOC-GM4

To understand the specificity of anionic **MOC** towards a binder, we have carried out gelation of **MOC** with mixture of binders. 0.01 M aqueous solutions of ammonium ions (NH_4^+), N-(2-aminoethyl)-1,3-propanediamine (AEPD), guanidine hydrochloride (gua.HCl), and β-alanine (β-ala) were prepared. The following combinations of binder solutions were added into **MOC** solution (20 mg of **1**in 1 ml water).

i) 100 µl NH_4^+ + 100 µl AEPD + 100 µlgua.HCl
ii) 100 µl AEPD + 100 µlgua.HCl + 100 µl β-ala
iii) 100 µl NH_4^+ + 100 µlgua.HCl + 100 µl β-ala
iv) 100 µl NH_4^+ + 100 µl AEPD + 100 µl β-ala

The mixtures were sonicated and kept at room temperature. In all cases opaque hydrogels are formed after 4-5 hours. The formation of hydrogel is confirmed by inversion test method. The hydrogels are named as i) **MOC-GM1**, ii) **MOC-GM2**, iii) **MOC-GM3**, and iv) **MOC-GM4.** The hydrogels are characterized by FESEM analysis.

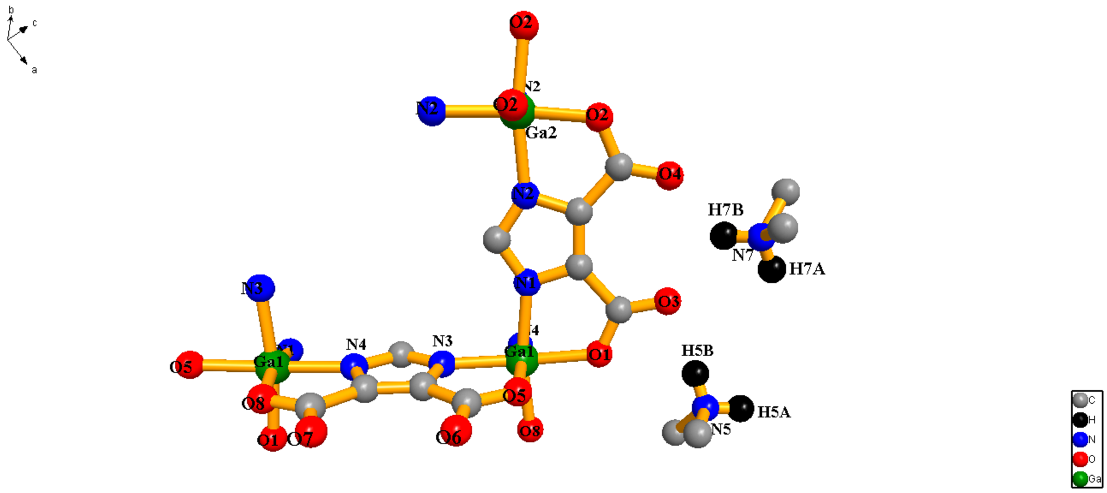

Supplementary Figure 4 | Asymmetric unit of 1. Asymmetric unit of **1** containing two Ga^{3+} (Ga1, Ga2) centres, two $ImDC^{3-}$, two dimethyl ammonium cations (Me_2NH^{2+}, DMA). Guest water and DMF molecules are deleted for clarity. Site occupancy of Ga1 = 1.0 and Ga2 = 0.33, Z = 3.

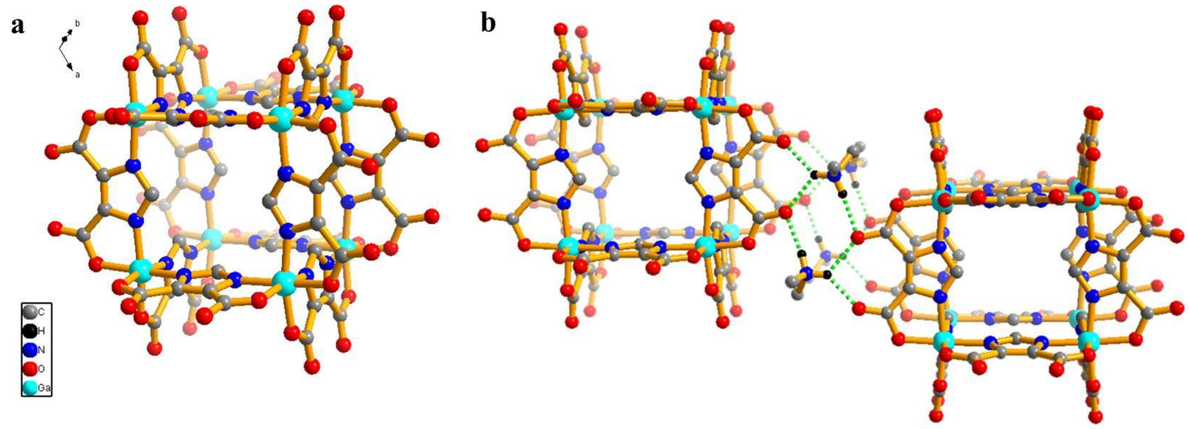

Supplementary Figure 5 | Crystal structure of MOC and intermolecular H-bonding between two MOCs. a, Structure of $[Ga_8(ImDC)_{12}]^{12-}$ cube. **b**, two $[Ga_8(ImDC)_{12}]^{12-}$ cube are connected to each other via intermolecular H-bonding with four DMA cations.

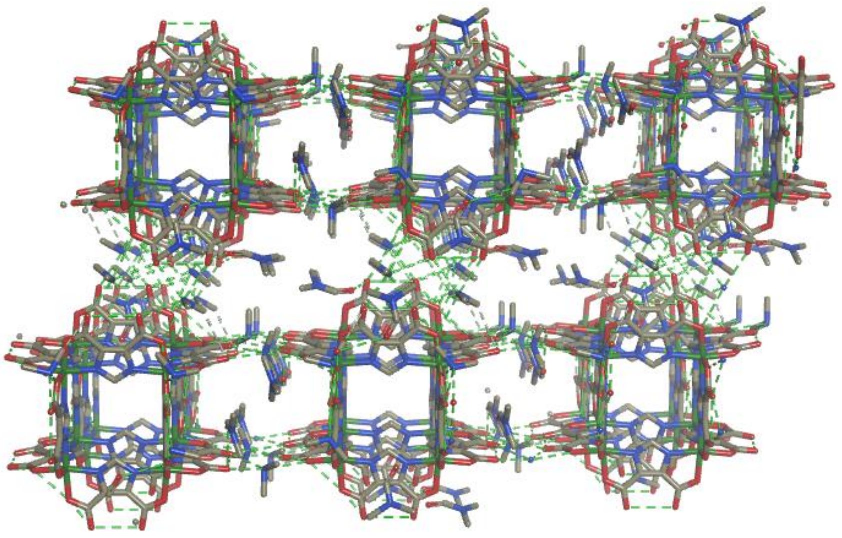

Supplementary Figure 6│Extended H-bonded crystal structure of 1. The 3D channels of **1** showing the presence of guest DMF and the structure is extended by the H-bonded DMA cations (water molecules are omitted for clarity).

Supplementary Table 1│Cell parameters and structure refinement parameters for1.

Parameter	1
Empirical formula	C_{87} H_{173} Ga_8 N_{37} O_{78}
Formula weight	3535.48
Crystal system	Trigonal
Space group	*R-3* (No.148)
a, Å	26.96000
b, Å	26.96000
c, Å	22.676(5)
γ, deg	120
V, Å^3	14274(4)
Z	3
T, K	294
μ,mm^{-1}	1.205
D_{calcd},g/cm^3	1.281
F (000)	5664
reflections [$I>2\sigma(I)$]	3358
unique reflections	6281
measured reflections	109182
R_{int}	0.110
GOF on F^2	1.11
$^aR_1[I>2\sigma(I)]$	0.0667
$R_w[I>2\sigma(I)]$	0.2640

$^aR_l= \sum||F_o|-|F_c||/\sum|F_o|; ^bR_w= [\sum\{w(F_o^2-F_c^2)^2\}/\sum\{w(F_o^2)^2\}]^{1/2}$

Supplementary Table 2 | Selected bond distances (Å) and bond angles (°) for 1.

Ga1-O1	1.980(5)	Ga2-O2	1.970(5)
Ga1-O5	1.980(5)	Ga2-N2	2.023(6)
Ga1-N1	2.024(5)	Ga2-O2_a	1.969(6)
Ga1-N3	2.030(5)	Ga2-N2_a	2.023(6)
Ga1-O8_e	1.976(8)	Ga2-O2_b	1.970(5)
Ga1-N4_e	2.034(5)	Ga2-N2_b	2.023(6)
O1-Ga1-O5	89.8(2)	O2_a-Ga2-N2	168.5(2)
O1-Ga1-N1	80.4(2)	N2-Ga2-N2_a	95.0(2)
O1-Ga1-N3	169.23(19)	O2_b-Ga2-N2	96.0(3)
O1-Ga1-O8_e	89.6(2)	N2-Ga2-N2_b	95.0(3)
O1-Ga1-N4_e	95.4(2)	O2_a-Ga2-N2_a	80.8(2)
O5-Ga1-N1	96.5(2)	O2_a-Ga2-O2_b	89.0(3)
O5-Ga1-N3	81.2(2)	O2_a-Ga2-N2_b	96.0(3)
O5-Ga1-O8_e	88.9(2)	O2_b-Ga2-N2_a	168.5(3)
O5-Ga1-N4_e	168.4(2)	N2_a-Ga2-N2_b	95.0(2)
N1-Ga1-N3	94.8(2)	O2_b-Ga2-N2_b	80.8(2)
O8_e-Ga1-N1	168.6(2)	Ga1-O1-C4	118.0(5)
N1-Ga1-N4_e	94.7(2)	Ga2-O2-C5	117.7(4)
O8_e-Ga1-N3	96.0(2)	Ga1-O5-C9	117.1(5)
N3-Ga1-N4_e	94.6(2)	Ga1_d-O8-C10	117.2(4)
O8_e -Ga1-N4_e	80.8(3)	Ga1-N1-C1	140.7(4)
O2-Ga2-N2	80.7(2)	Ga1-N1-C3	112.4(5)
O2-Ga2 -O2_a	89.0(2)	Ga2-N2-C1	141.9(5)
O2-Ga2-N2_a	96.0(2)	Ga2-N2-C2	112.1(4)
O2-Ga2-O2_b	89.0(2)	Ga1-N3-C6	142.1(4)
O2-Ga2-N2_b	168.5(2)	Ga1-N3-C7	111.2(4)

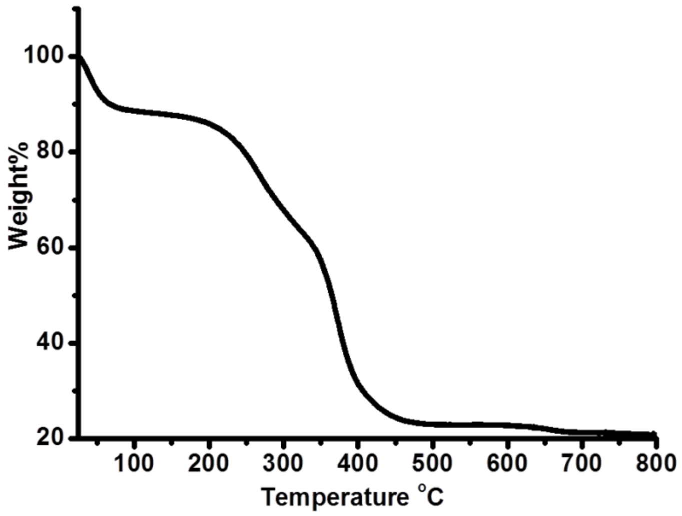

Supplementary Figure 7│Thermal stability of 1. TGA profile of **1** in temperature range 25°C – 800°C under nitrogen atmosphere.

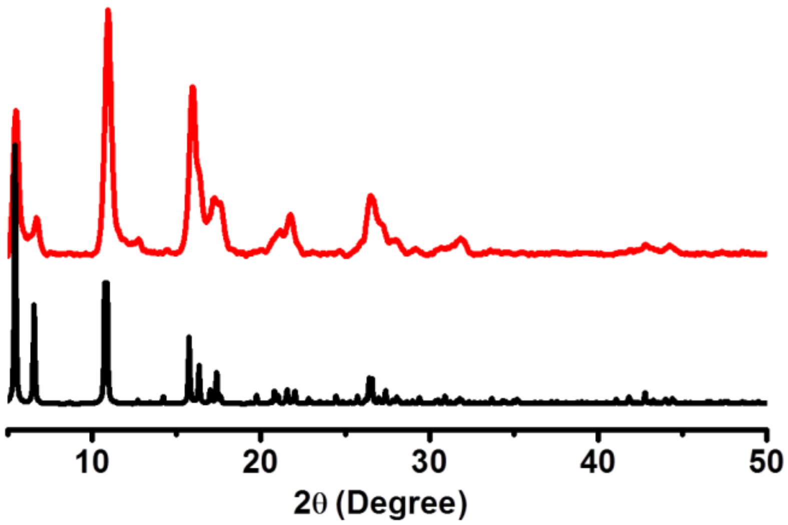

Supplementary Figure 8│Phase purity of 1. PXRD patterns of **1** simulated (black) and as-synthesized (red). The similarity in PXRD pattern indicates phase purity of the powder sample of **1**.

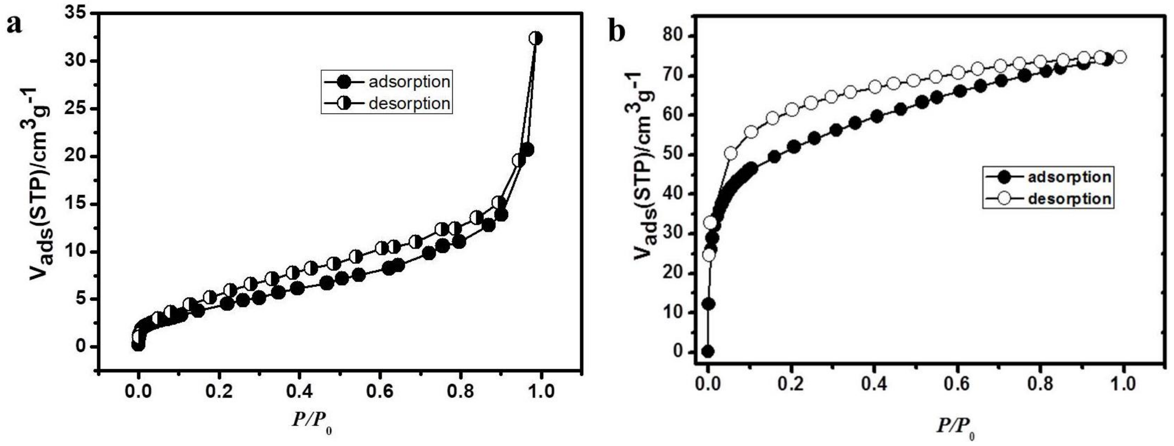

Supplementary Figure 9│Gas adsorption measurements of 1. a, N_2 adsorption profile of **1** at 77 K. **b**,CO_2 adsorption profile of **1** at 195 K.

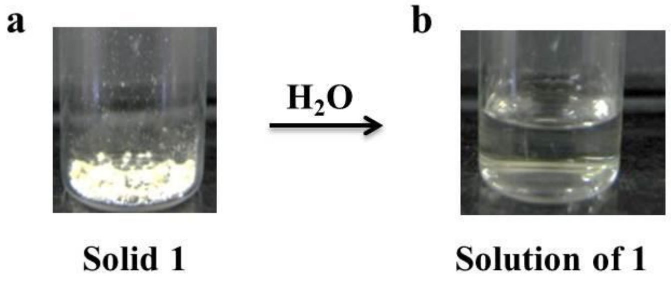

Supplementary Figure 10│Solubility of 1 in water. a, Picture of solid powder of **1**. **b**, Picture of the aqueous solution of **1.** The solid powder of **1** gets completely dissolve in water.

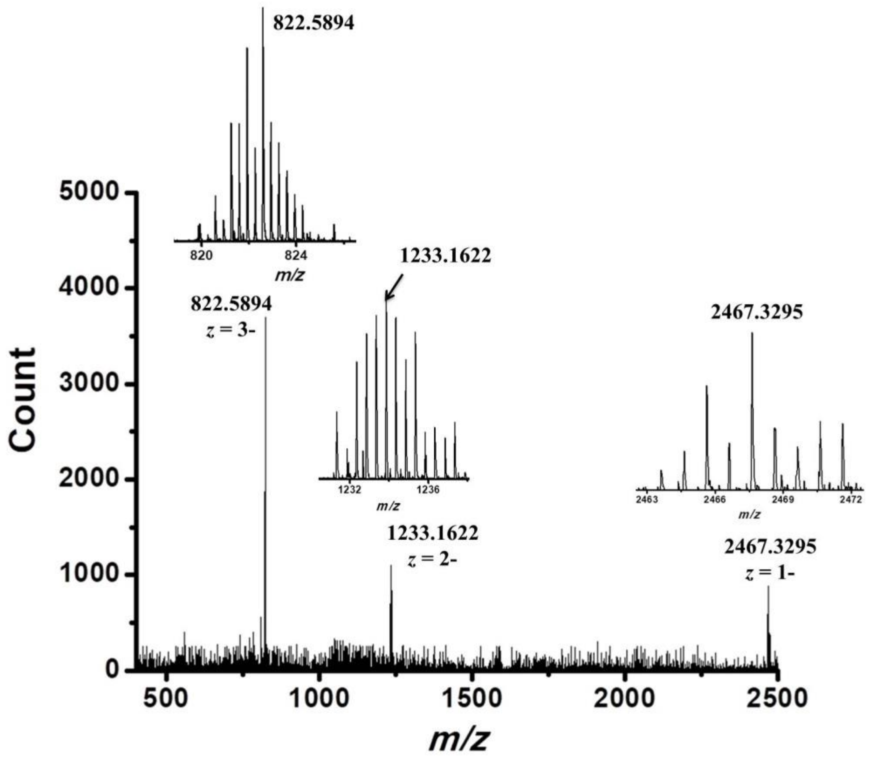

Supplementary Figure 11 │ HRMS of aqueous solution of 1. The negative mode acquisition mode HRMS of the aqueous solution of **1** showing the peak at *m/z*= 2467.3295 (z=1^-), 1233.1622 (z=2^-) and 822.5894 (z=3^-) corresponding to $[\{Ga_8(ImDC)_{12}\}\{9H^+\}\{2Na^+\}\{H_2O\}]^-$, $[\{Ga_8(ImDC)_{12}\}\{8H^+\}\{2Na^+\}\{H_2O\}]^{2-}$, and $[\{Ga_8(ImDC)_{12}\}\{7H^+\}\{2Na^+\}\{H_2O\}]^{3-}$ moieties, respectively.

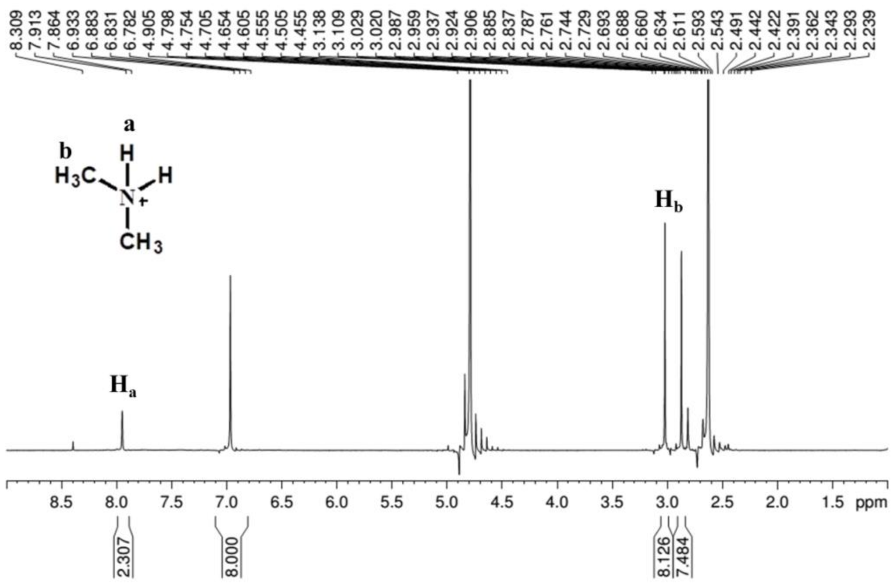

Supplementary Figure 12 │ ^{1}H-NMR of 1. ^{1}H-NMR spectrum of **1** after dissolving in D_2O.

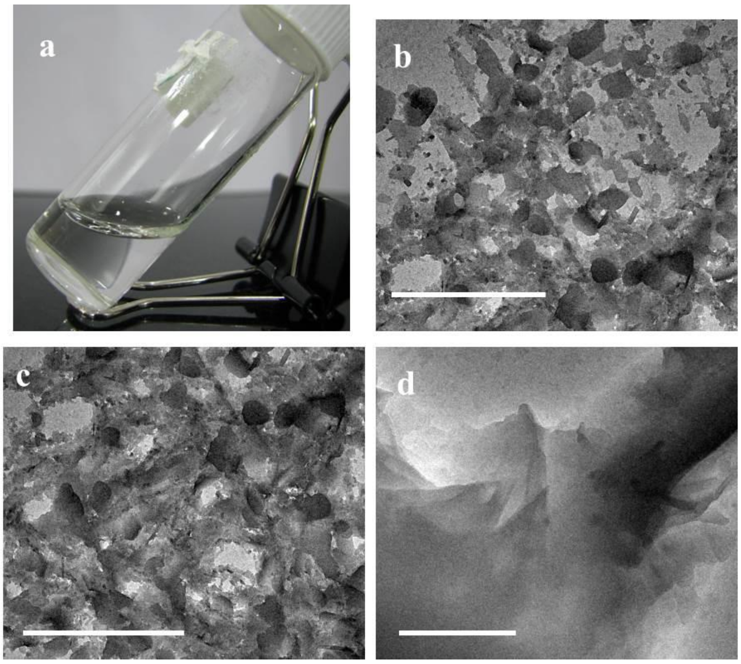

Supplementary Figure 13│No self-assembly of 4,5-imidazoledicarboxylic acid with ammonium cation. **a**, Photograph of aq. solution of 4,5-imidazoledicarboxylic acid containing NH_4^+ cations as molecular binders. The solution does not form gel even after several weeks. **b-d**, TEM images of the same solution showing presence of random flacks.**a**, **b**, Scale bar = 2 μm and **c**, scale bar = 1 μm.

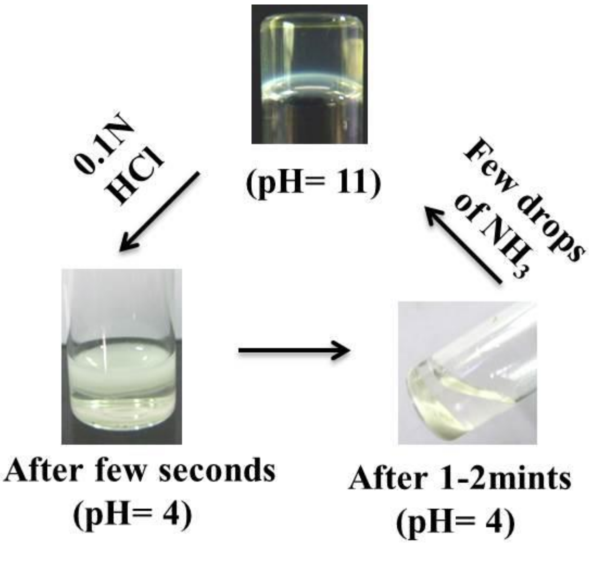

Supplementary Figure 14│pH responsive behaviour of MOC-G1 hydrogel. When 0.1 N HCl (pH= 4-5) is added to **MOC-G1** (intrinsic pH =11) a precipitate forms which reforms hydrogel after addition of aq. NH_3 (pH = 12).

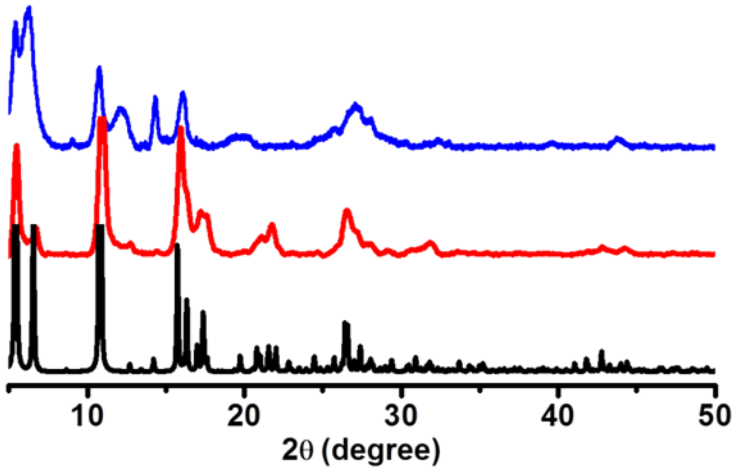

Supplementary Figure 15│Comparison of PXRD patterns.PXRD pattern of simulated **1**(black), as-synthesized **1** (red) and **MOC-G1** xerogel (blue). Presence of similar Bragg's reflections indicates stability of **MOC**s in **MOC-G1** gel.

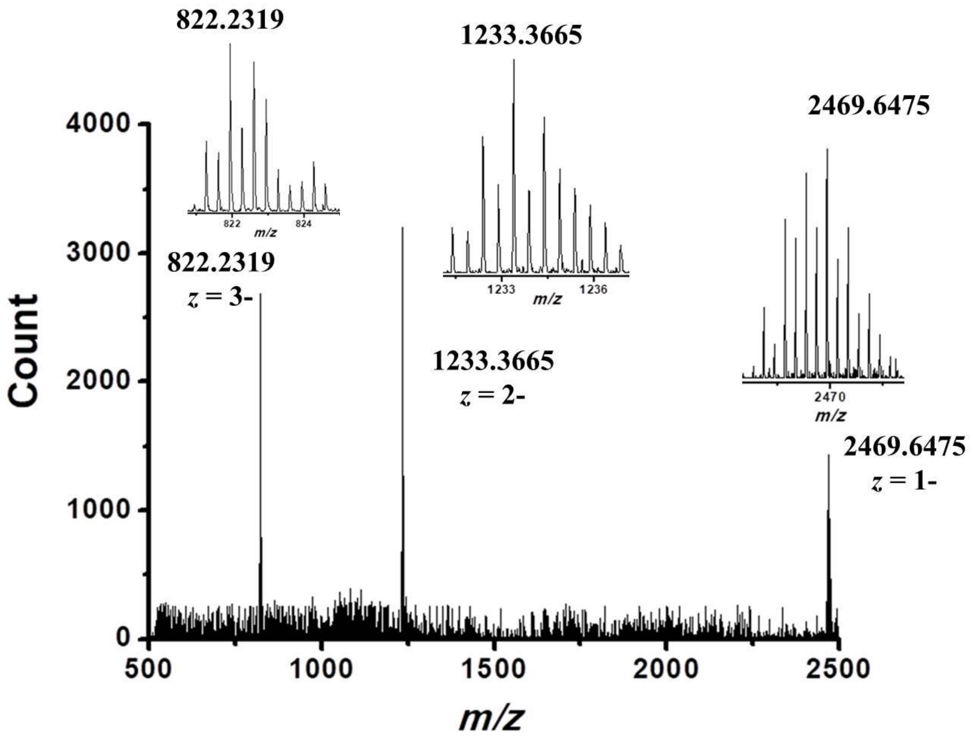

Supplementary Figure 16│HRMS of MOC-G1. Negative mode HRMS of the aqueous solution of **MOC-G1** xerogel showing peaks at m/z= 2469.6475 (z = 1^-), 1233.3665 (z = 2^-) and 822.2319 (z = 3^-).

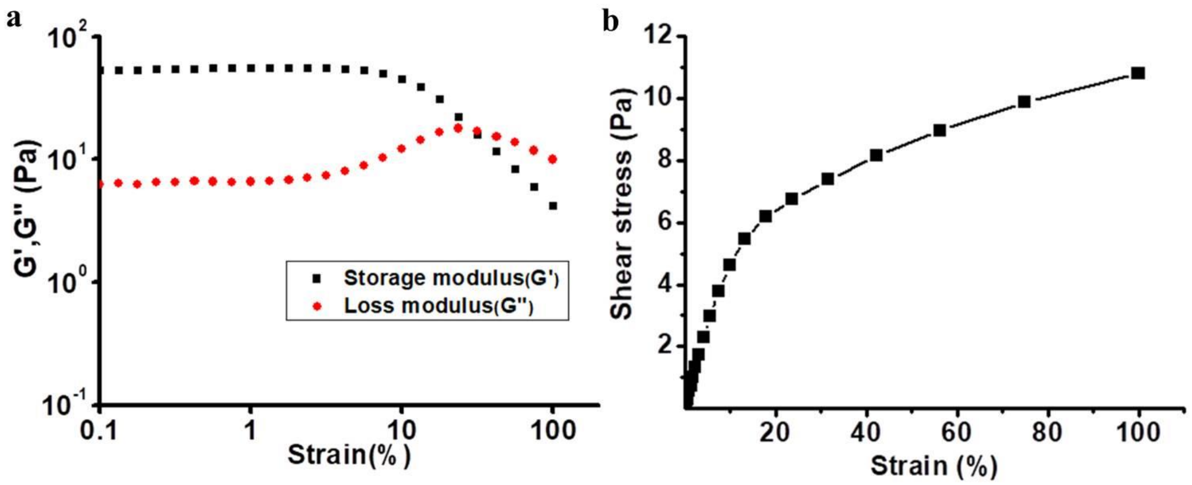

Supplementary Figure 17 | Rheological analysis of MOC-G1 hydrogel. a, Oscillatory strain measurements (frequency=1.0 rad s^{-1}) of **MOC-G1**, the squares (black) and circles (red) indicate storage (G') and loss modulus (G"), respectively. **b**, The stress *vs* stain plot of **MOC-G1** hydrogel.

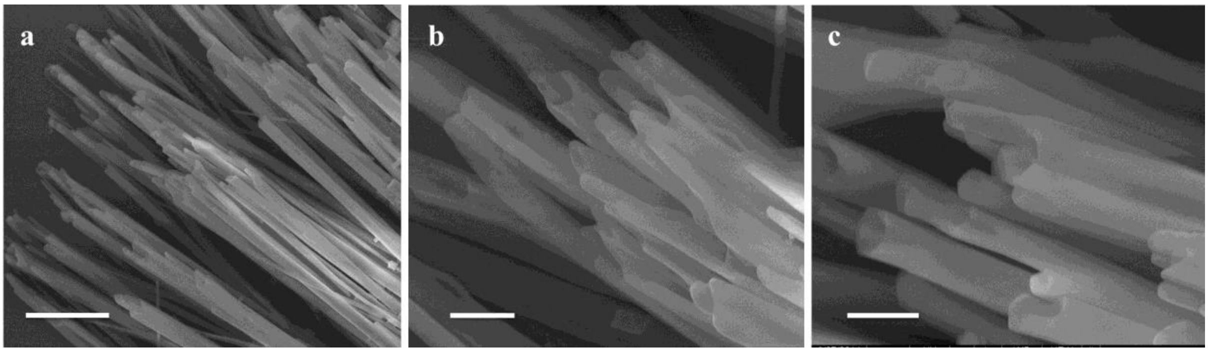

Supplementary Figure 18 | FESEM images of MOC-G1 xerogel. FESEM images of **MOC-G1** xerogel showing nanotube morphology with rectangular cross-section. **a**, Scale bar = 5μm and **b**, **c**, scale bar = 1μm.

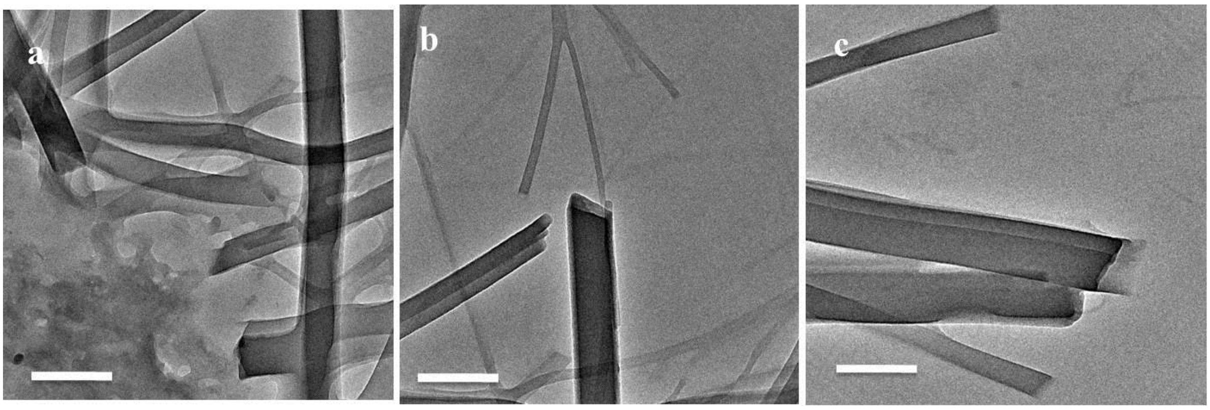

Supplementary Figure 19│TEM images of MOC-G1 xerogel.TEM images of **MOC-G1** xerogel showing the formation of nanotubes with rectangular cross-section. **a**, **b** and **c**, Scale bar = 200 nm.

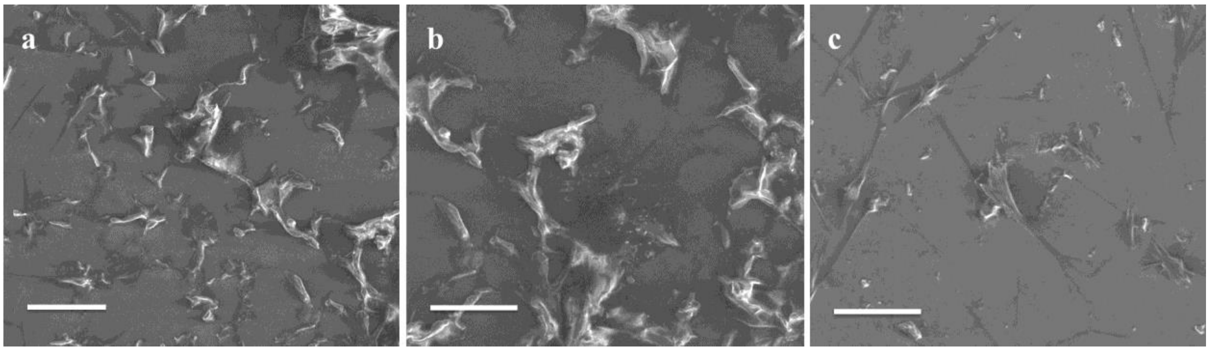

Supplementary Figure 20│ Time dependent morphology of MOC/NH_3 solution.FESEM images of **MOC**/NH_3 solution after 2 hours showing the formation of small crumpled sheet. **a**, Scale bar = 50 μm. **b** and **c**, Scale bar = 40 μm.

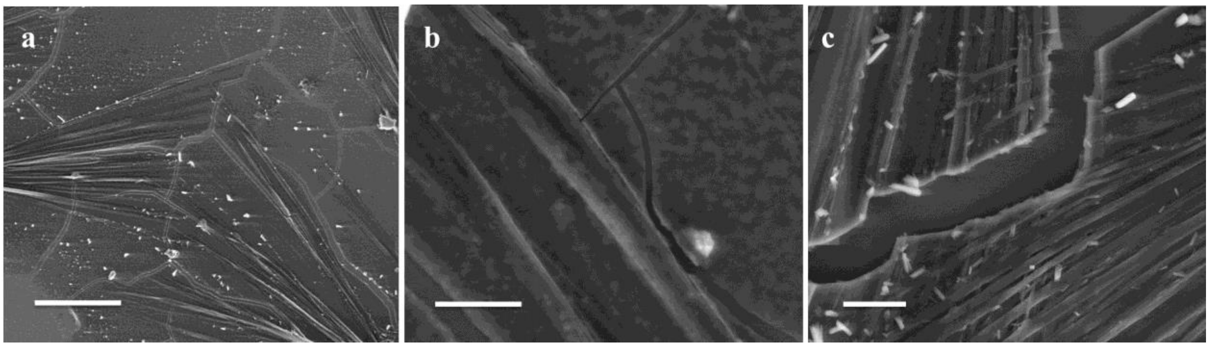

Supplementary Figure 21│ Time dependent morphology of MOC/NH_3 solution.FESEM images of **MOC**/NH_3 solution after 4 hours showing the formation of micron size tapes. **a**, Scale bar = 30 μm. **b**, Scale bar = 4 μm and **c**, Scale bar = 5 μm.

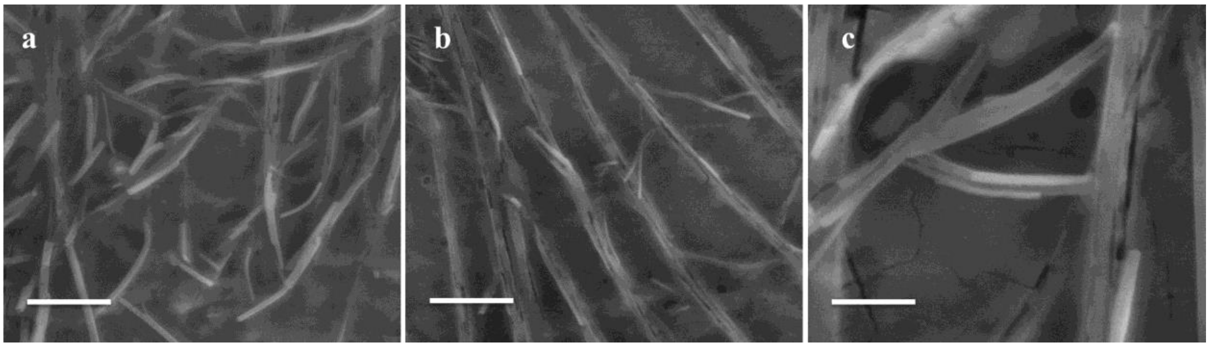

Supplementary Figure 22│ Time dependent morphology of MOC/NH_3 solution. FESEM images of **MOC**/NH_3 solution after 6 hours showing the formation of partially formed tubes. **a**, **b**, Scale bar = 2 μm and **c**, Scale bar = 1 μm.

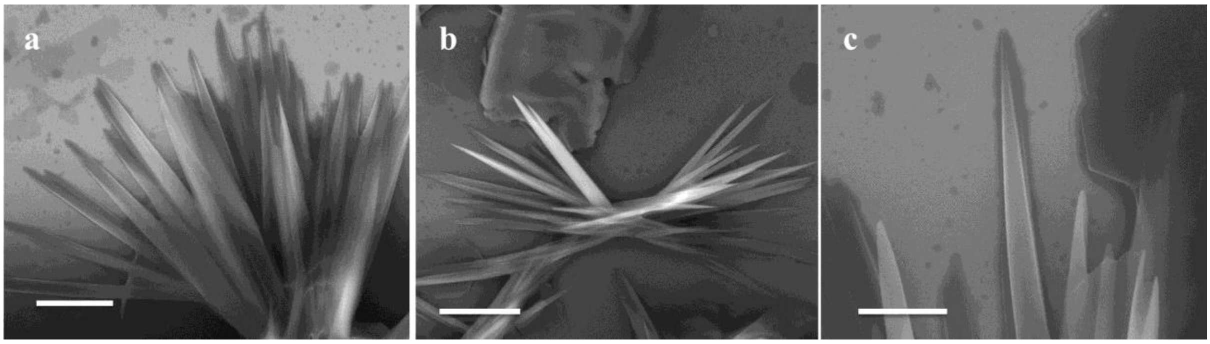

Supplementary Figure 23│ FESEM images of MOC-G2 xerogel. FESEM images of **MOC-G2** xerogel showing the formation of bouquet-like nanostructures. **a**, Scale bar = 2 μm. **b**, Scale bar = 5 μm and **c**, Scale bar = 1 μm.

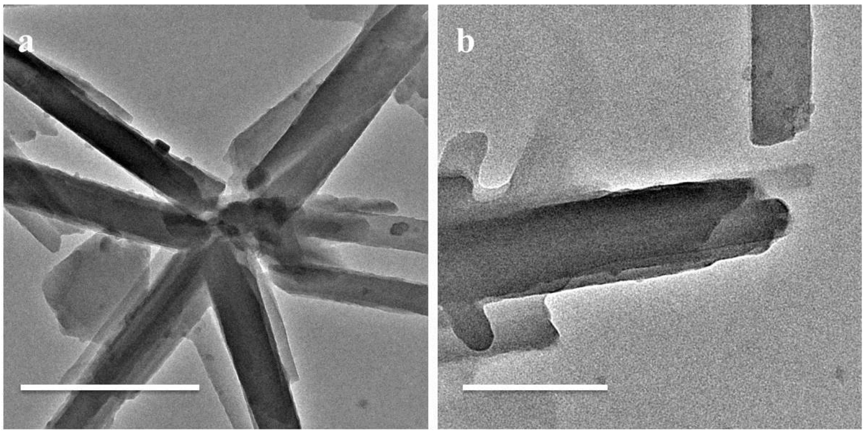

Supplementary Figure 24│TEM images of MOC-G2 xerogel. TEM images of **MOC-G2** xerogel showing the formation of bouquet-like nanostructures. **a**, Scale bar = 500 nm and**b**, Scale bar = 200 μm.

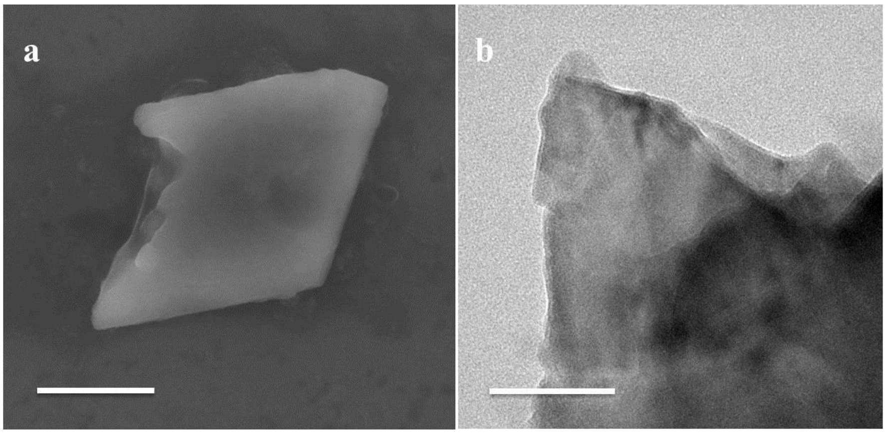

Supplementary Figure 25│ Morphology of MOC-G3 xerogel. **a**, FESEM image and **b**, TEM image of **MOC-G3** xerogel showing the formation of sheet-like morphology. **a**, Scale bar = 1 μm and **b**, scale bar = 200 nm.

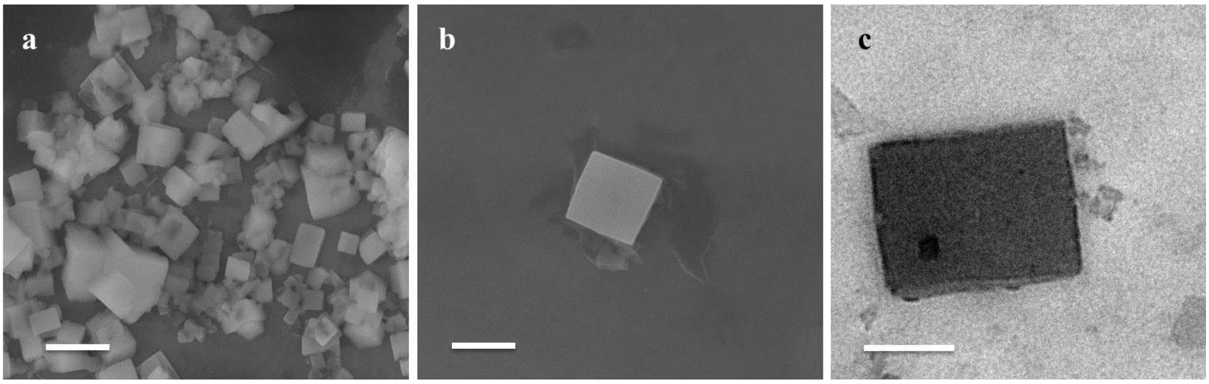

Supplementary Figure 26│ Morphology of MOC-G4 xerogel. **a** and **b**, FESEM images of **MOC-G4** xerogel. **c**, TEM images of **MOC-G4** xerogel showing the formation of nanocubes. **a**, Scale bar = 1μm. **b**, Scale bar = 500 nm and **c**, scale bar = 200 nm.

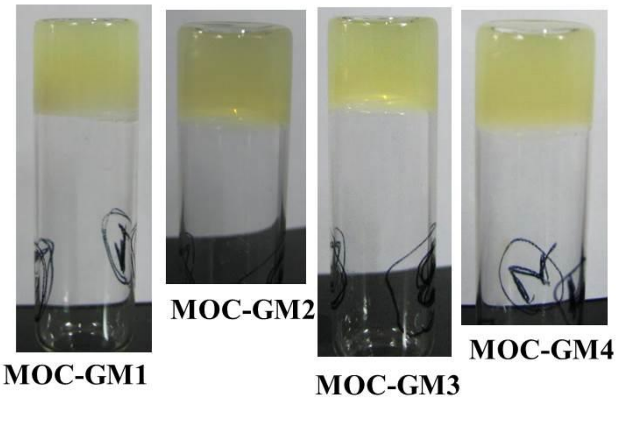

Supplementary Figure 27│ Images of mixed-binder hydrogels. Photograph of **MOC-GM1**, **MOC-GM2**, **MOC-GM3** and **MOC-GM4**, respectively.

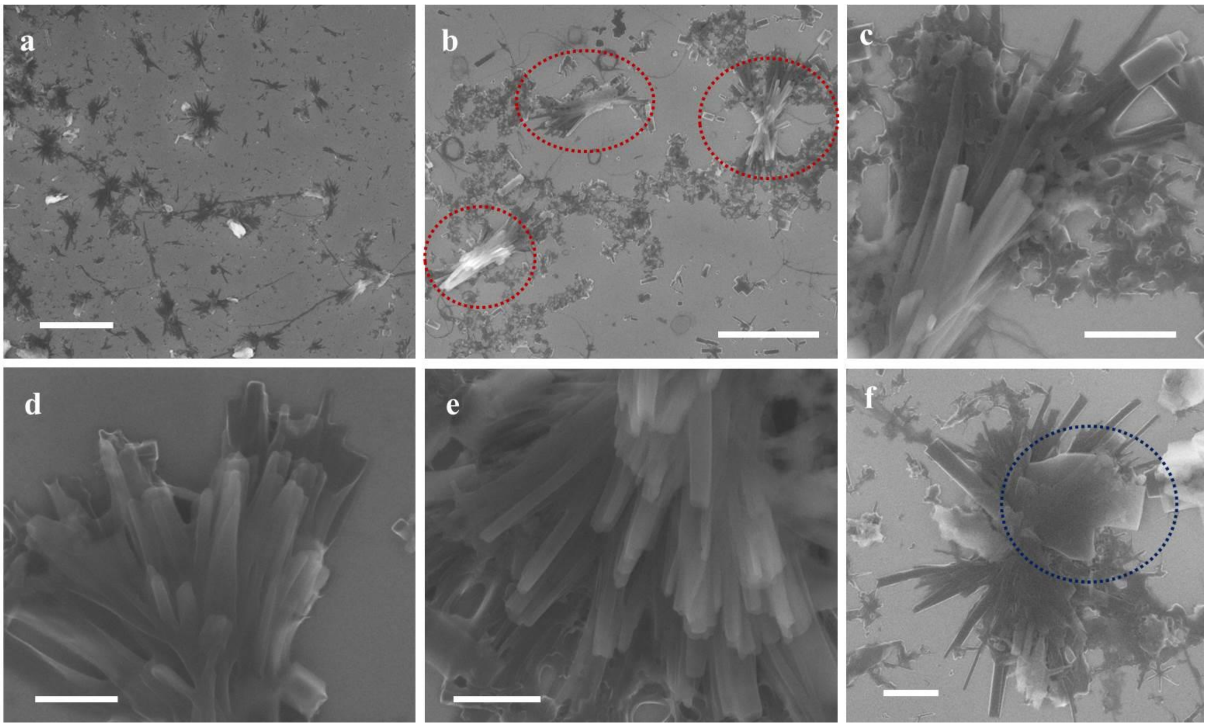

Supplementary Figure 28│Mixture of morphologies in MOC-GM1 xerogel. a, FESEM images of **MOC-GM1** xerogel showing co-existence of nanotubes, needle-*like* and sheet-*like* nanostructures distributed over a large area. **b**, FESEM image of **MOC-GM1** xerogel. The red circles highlight bunches of nanotubes and needle-*like* nanostructures. **c-e**, FESEM images of **MOC-GM1** xerogel showing the presence of nanotubes and needle-*like* nanostructures. **f**, FESEM image of**MOC-GM1** xerogel showing co-existence of sheet-like and needle-*like* nanostructures. The blue circle highlights the presence of nano-sheet.**a**, Scale bar = 5μm. **b**, Scale bar = 3 μm. **c**, Scale bar = 500 nm. **d**, Scale bar = 300 nm. **e**, Scale bar = 400 nm and **f**, scale bar = 500 nm.

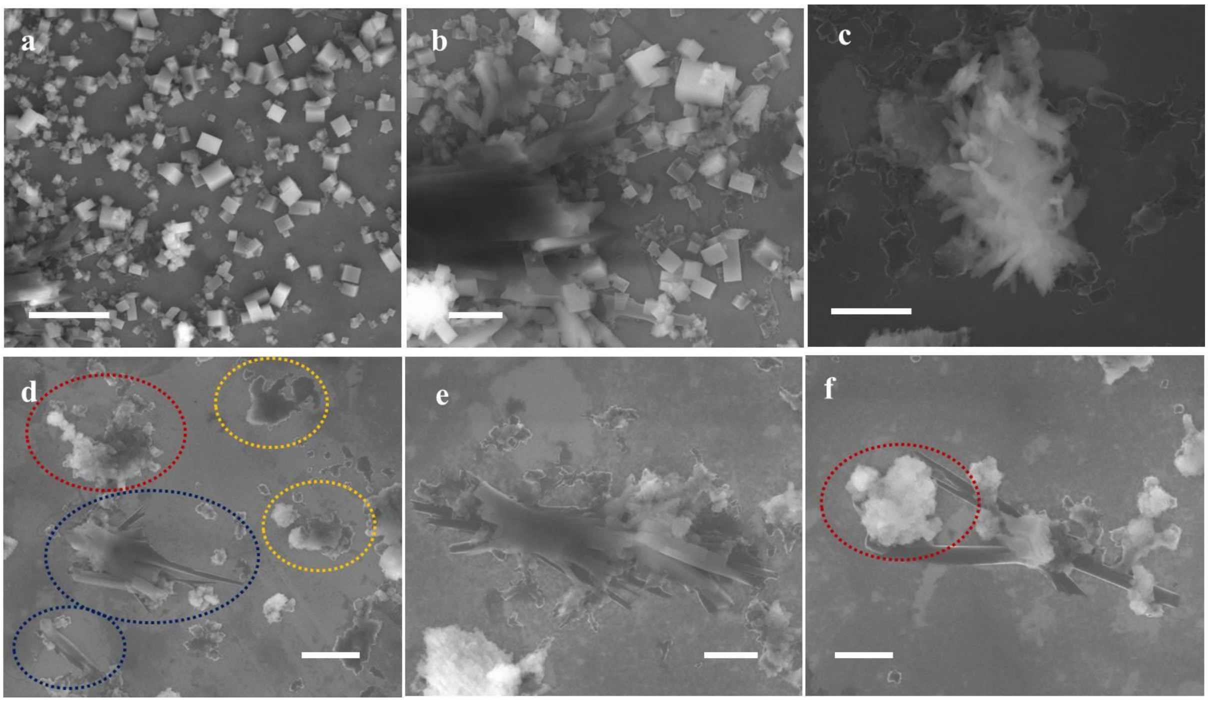

Supplementary Figure 29 | Mixture of morphologies in MOC-GM2 xerogel. a-b, FESEM images of **MOC-GM2** xerogel showing the presence of nanocubes over a large area. **c**, FESEM image of **MOC-GM2** xerogel showing the presence of needle-*like* nanostructures. **d**, FESEM image of **MOC-GM2** xerogel showing the co-existence of needle-*like* nanostructures (blue circle), nanocubes (red circle) and sheet-like nanostructures (yellow circle), **e-f**, Showing the presence of needle-*like* nanostructures and nanocubes. The red circle highlights the nanocubes. **a**, Scale bar = 3µm. **b**, **c**, **d**, Scale bar = 1 µm. **e**, **f**, Scale bar = 500 nm.

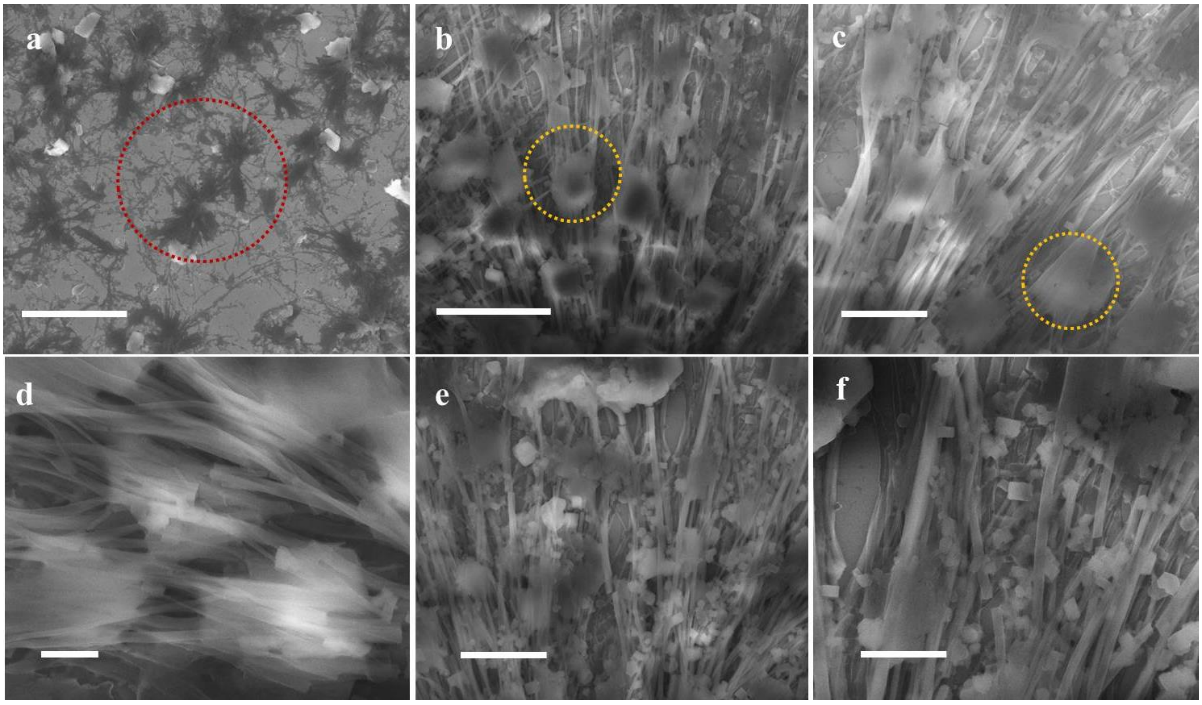

Supplementary Figure 30│ Mixture of morphologies in MOC-GM3 xerogel. a, FESEM image of **MOC-GM3** xerogel showing the bunches of nanotubes (highlighted by red circle) distributed over a large area. **b-d**, FESEM images of **MOC-GM3** xerogel showing co-existence of nanotubes and nanosheets. Nanosheets are highlighted by yellow circles. **e-f**, FESEM images of **MOC-GM3** xerogel showing co-existence of nanotubes and nanocube. **a**, Scale bar = 5µm. **b**, Scale bar = 4 µm. **c**, Scale bar = 2 µm. **d**, Scale bar = 500 nm. **e**, Scale bar = 2µm and **f**, scale bar = 1 µm.

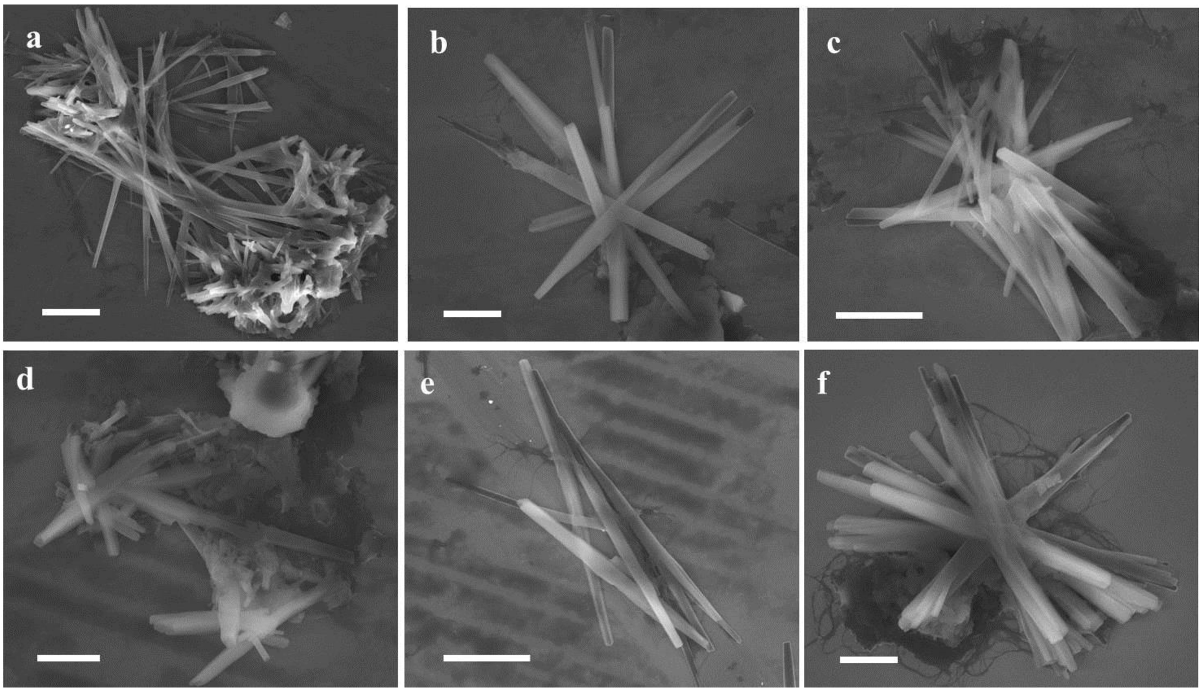

Supplementary Figure 31 | Mixture of morphologies in MOC-GM4 xerogel. a-f, FESEM images of **MOC-GM4** xerogel showing the co-existence of nanotube and needle-*like* nanostructures. Very few numbers of nanocubes are also present. **a**, Scale bar = 10μm. **b**, Scale bar = 1 μm. **c**, Scale bar = 2 μm. **d**, Scale bar = 1 μm. **e**, Scale bar = 2μm and **f**, scale bar = 1 μm.

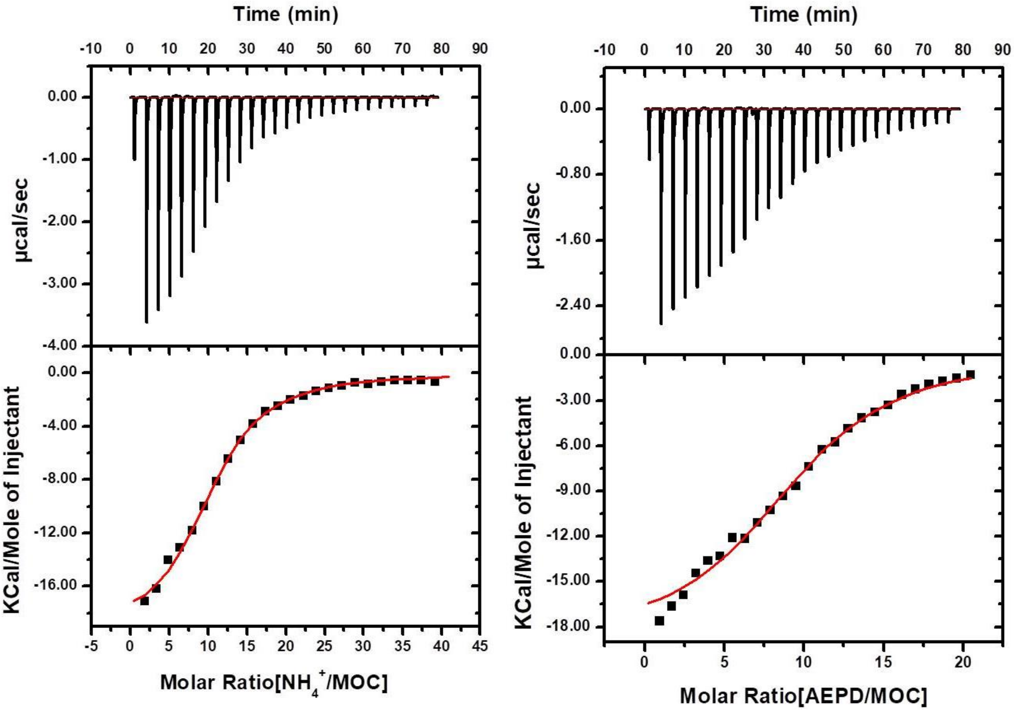

Supplementary Figure 32│Determination of binding affinity. Isothermal titration calorimetry (ITC) analysis of **MOC** (concentration = 50 μM) with**a**, NH_4^+ (Concentration = 2000 μM) and **b**, AEPD (concentration = 1000 μM) in water at 25 °C. The upper panel shows the raw data curve, and the lower panel shows the fitted integrated ITC data curve.

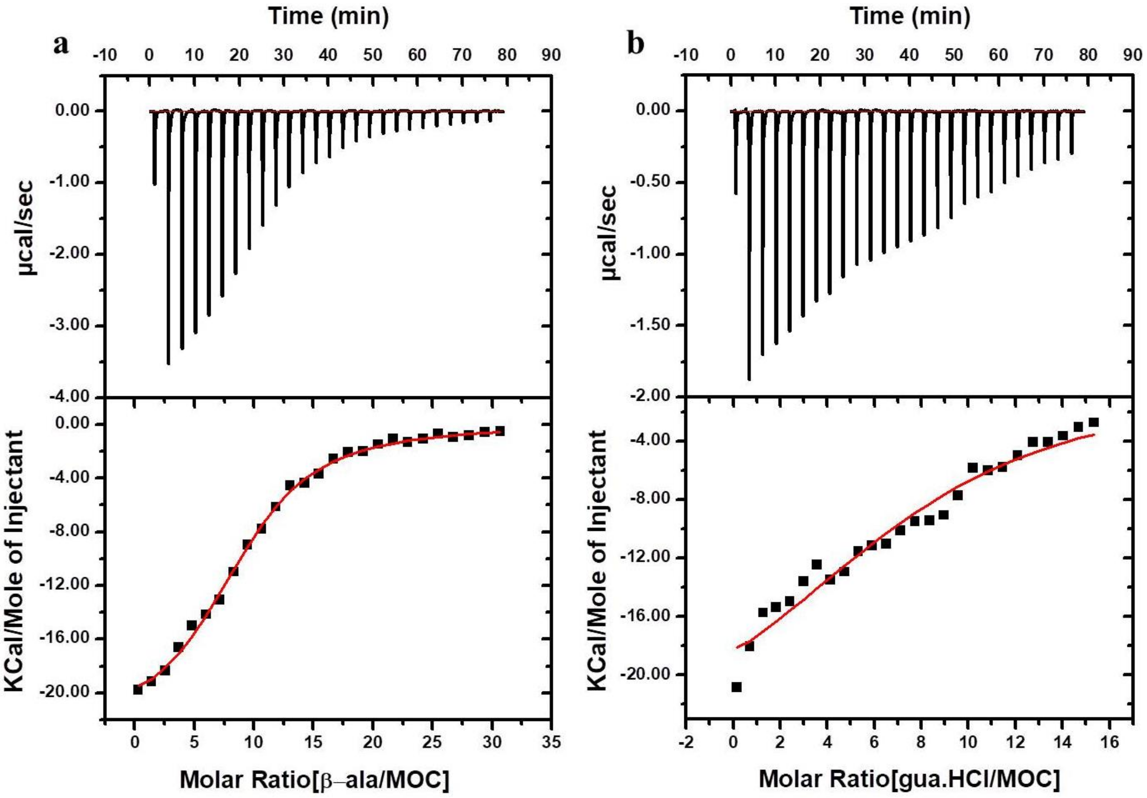

Supplementary Figure 33│ Determination of binding affinity. Isothermal titration calorimetry (ITC) analysis of **MOC** (concentration = 50 μM) with **a**, β-ala (concentration = 1500 μM) and **b**, gua.HCl (concentration = 750 μM) in water at 25 °C.The upper panel shows the raw data curve, and the lower panel shows the fitted integrated ITC data curve.

Supplementary Table 3│ The binding affinity values (K_a), stoichiometry (N), enthalpy (ΔH), entropy (ΔS) and free energy (ΔG) of different binders towards **MOC**.

Binder	K_a (M^{-1})	N	ΔH (kcal $mole^{-1}$)	TΔS (kcal $mole^{-1}$)	ΔG (kcal $mole^{-1}$)
NH_4^+	6.65×10^4	10.5	-19.61	-1.0925	-18.5175
AEPD	6.76×10^4	9.86	-18.90	-1.0325	-17.8675
β-ala	6.50×10^4	9.1	-22.86	-1.3650	-21.4950
Gua.HCl	2.73×10^4	7.96	-26.53	-1.7175	-24.8125

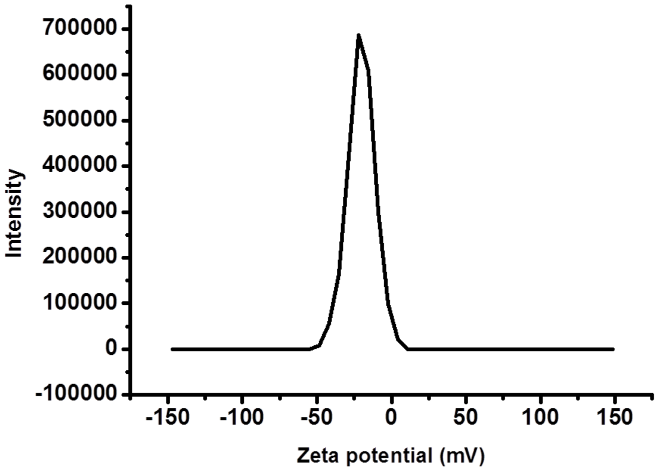

Supplementary Figure 34│Negatively charged surface of nanotubes. Zeta potential of **MOC-G1** xerogel (dispersed in MeOH) found to be -22 mV, indicating negatively charged surface of the nanotubes.

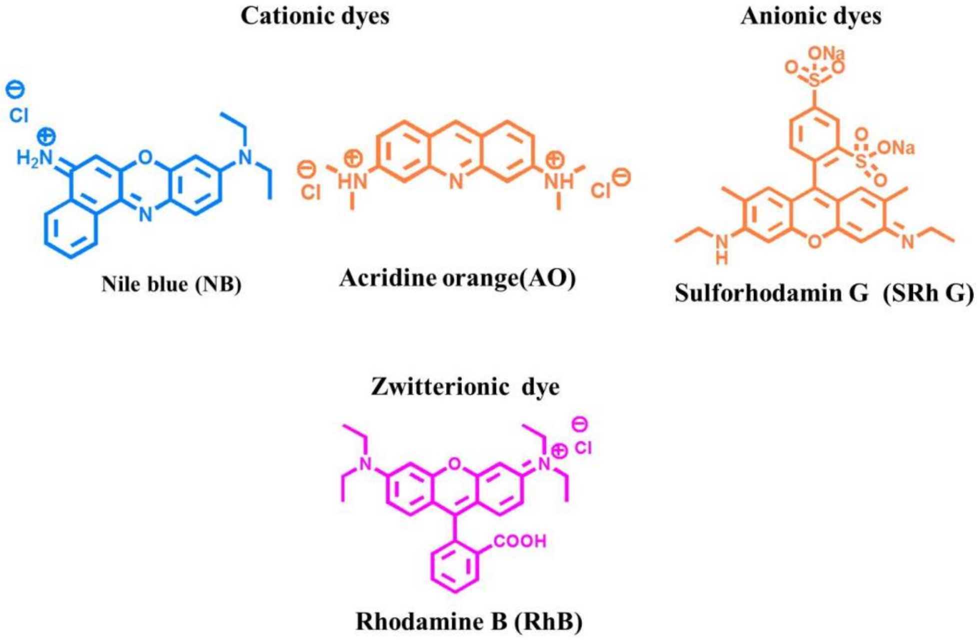

Supplementary Figure 35│ Dyes selected for demonstrating separation of charged species by MOC-G1 hydrogel. The molecular structures of cationic (nile blue, acridine orange), anionic (sulforhodamin G) and zwitterionic (rhodamine B) dyes.

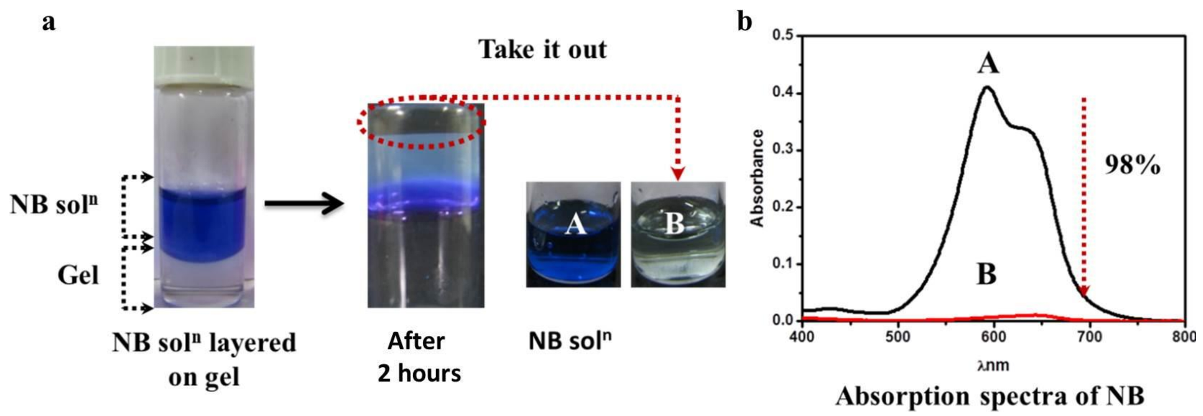

Supplementary Figure 36│Adsorption of nile blue by MOC-G1 hydrogel.a, 10^{-5}M aqueous solution of nile blue (**NB**) was layered on the **MOC-G1** gel. After few hours most of the **NB**gets absorbed in the gel.**A** is the 10^{-5}M**NB** solution.**B** is the clear solution collected below the gel after few hours. This solution was taken out by using a syringe.**b**, Absorption spectra of 10^{-5} M **NB** solution (black) and the clear solution (red) collected below the hydrogel.

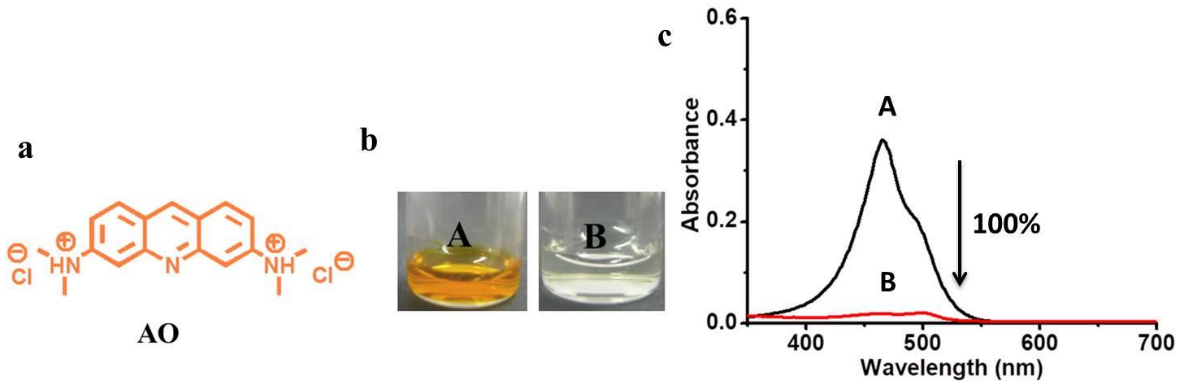

Supplementary Figure 37│Adsorption of acridine orange by MOC-G1 hydrogel. a,The molecular structure of acridine orange (**AO**). **b**, **A** is the 10^{-5} M **AO** solution which was layered above the **MOC-G1** hydrogel and **B** is the clear solution collected below the hydrogel after few hours. **c**, Absorption spectra of 10^{-5} M **AO** solution (black) and the clear solution (red) collected below the **MOC-G1** hydrogel.

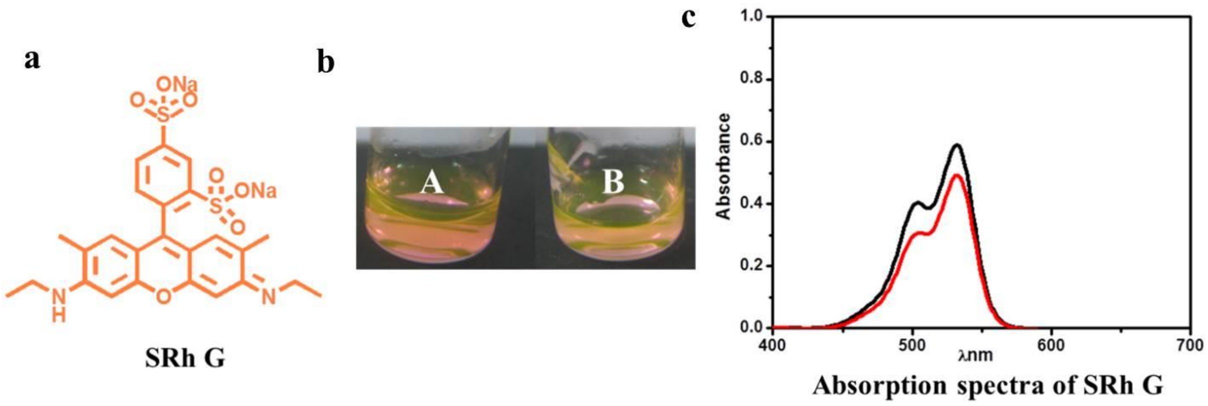

Supplementary Figure 38│ Adsorption of sulforhodamin G by MOC-G1 hydrogel. a, The molecular structure of sulforhodamin G (**SRh G**). **b**,**A** is the 10^{-5}M **SRh G**solution which was layered above the **MOC-G1** hydrogel and**B** is the solution collected below the gel after few hours. **c**, Absorption spectra of 10^{-5} M **SRh G**solution (black) and the solution (red) collected below the hydrogel.

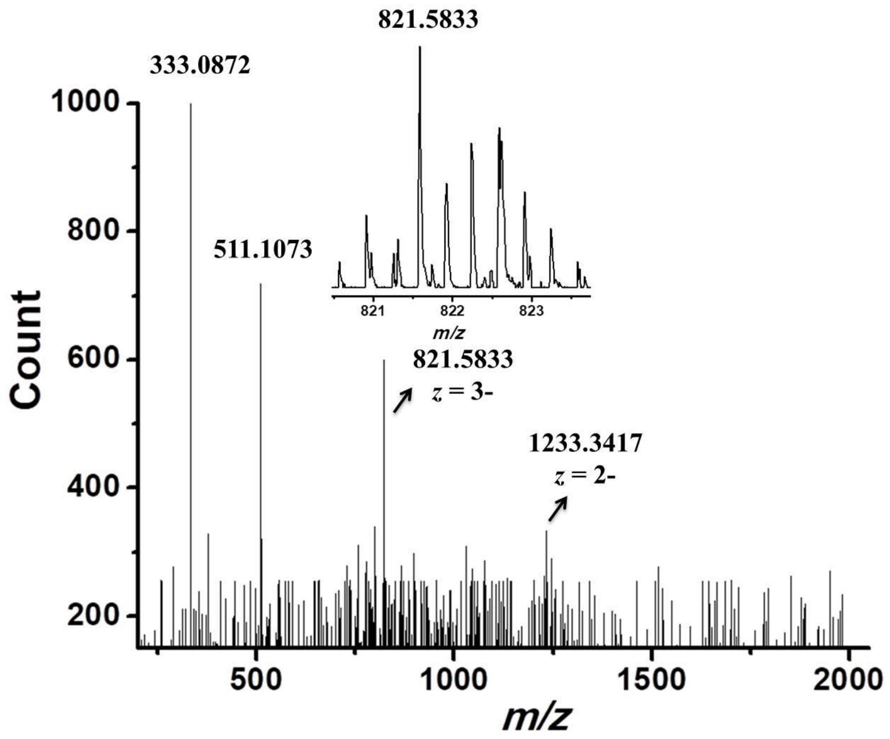

Supplementary Figure 39│ Stability of MOC after adsorption. HRMS of **NB** encapsulated **MOC-G1**(**NB@MOC-G1**) xerogel showing peaks at *m/z*= 821.5833 and 1233.3417, corresponding to corresponding to $[(2Na^{+})(7H^{+})\{Ga_8(ImDC)_{12}\}(H_2O)]^{3-}$ and $[(2Na^{+})(8H^{+})\{Ga_8(ImDC)_{12}\}(H_2O)]^{2-}$ moieties, respectively. This indicates stability of **MOC** in **NB@MOC-G1**.

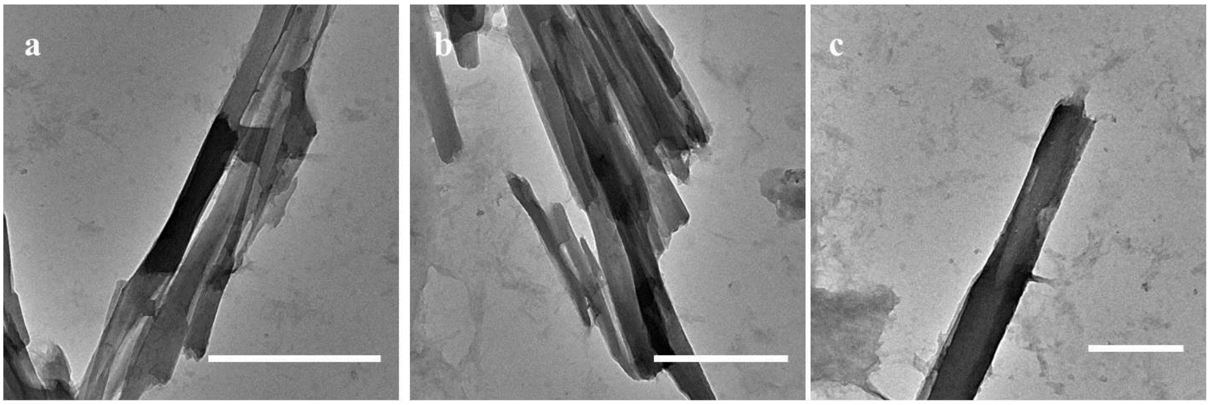

Supplementary Figure 40│ Nanotube morphology of NB@MOC-G1 xerogel. The TEM images of the **NB@MOC-G1**showing nanotube morphology. This indicates that adsorption of dye does not change the morphology of **MOC-G1** hydrogel.**a**, Scale bar = 1 μm. **b** and **c**, Scale bar = 500 nm.

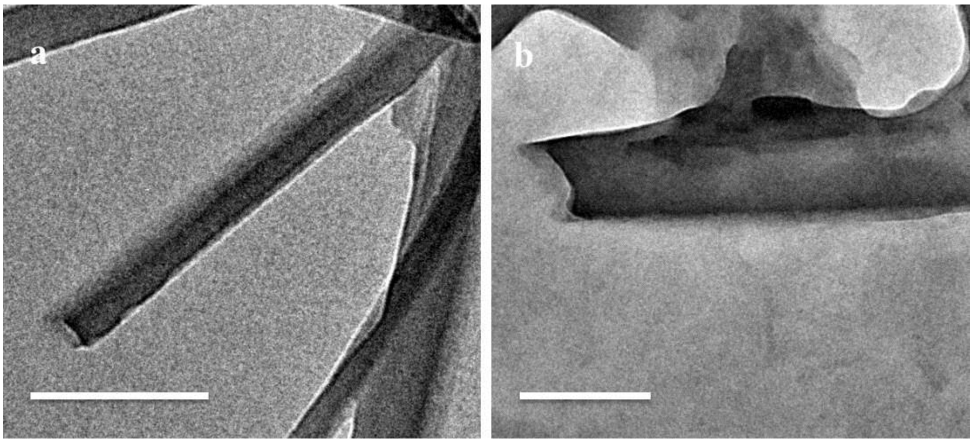

Supplementary Figure 41│ Retention of nanotube morphology after washing. The TEM images of the **NB@MOC-G1** after repeated MeOH wash. Retention of nanotube morphology indicates stability of nanotubes after the removal of adsorbed dye.**a**, Scale bar = 500 nm and **b**, scale bar = 200nm.

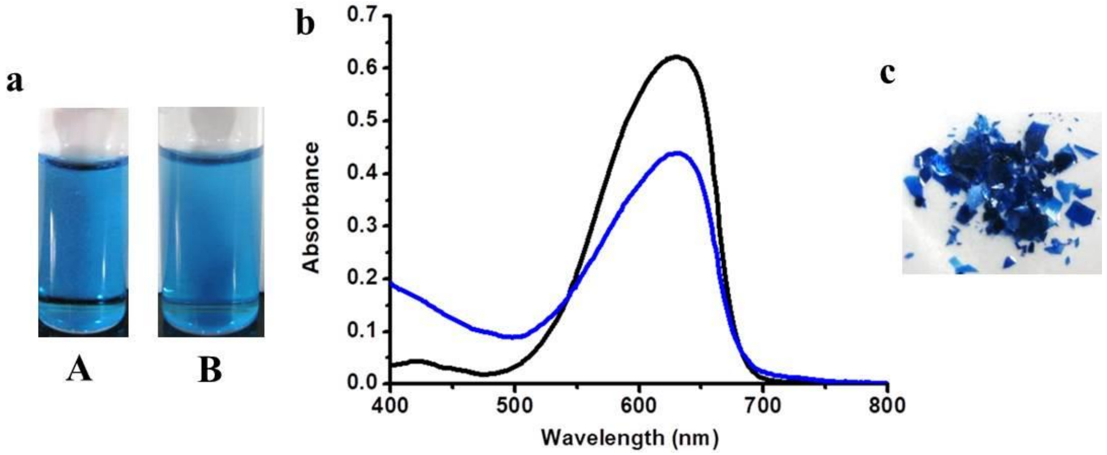

Supplementary Figure 42│ Adsorption of nile blue by 1. a, A is the 10^{-5} M methanolic solution of Nile blue and B is the solution of nile blue after soaking the crystals of **1** into it for 2 days. **b**,Absorption spectra of 10^{-5} M nile blue solution (black) and the solution (blue) obtained after soaking the crystals of **1**.**c**,The picture of the crystals of **1**after soaking it in the nile blue solution for 2days and washing repeatedly by methanol.

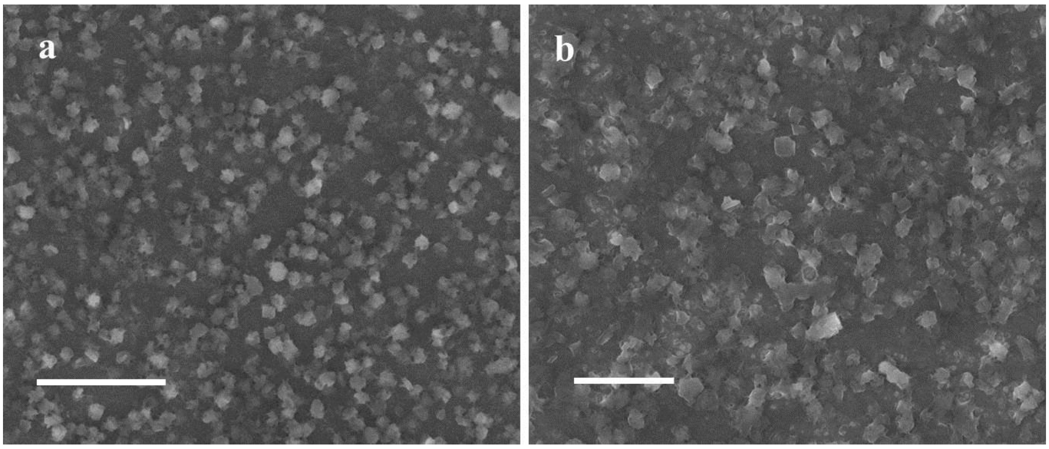

Supplementary Figure 43│ Formation of MOC cubes after precipitation. The FESEM images of the precipitate formed after addition of 0.1 N HCl to the **NB@MOC-G1. a**, Scale bar = 3 μm and **b**, scale bar = 2 μm.

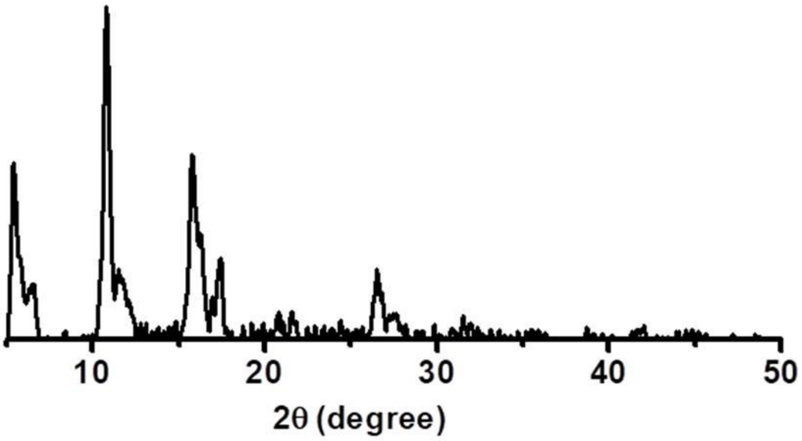

Supplementary Figure 44 | Stability of MOC after precipitation. The PXRD pattern of the precipitate formed after addition of 0.1N aq. HCl into the **NB@MOC-G1** hydrogel.

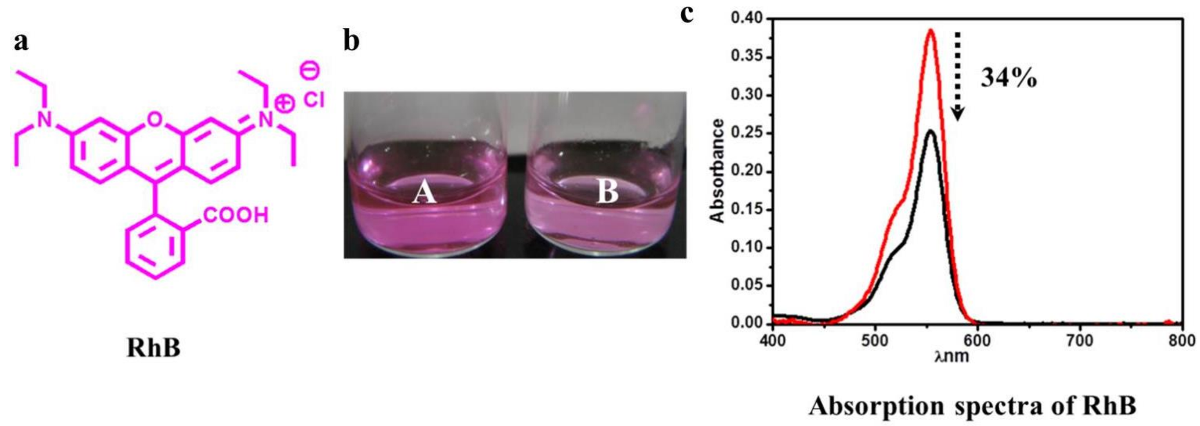

Supplementary Figure 45 | Adsorption of rhodamine B by MOC-G1 hydrogel. a,The molecular structure of rhodamine B (**RhB**). **b**, A is the 10^{-5}M **RhB** solution which was layered above the **MOC-G1** hydrogel and B is the solution collected below the hydrogel after few hours. **c**, Absorption spectra of 10^{-5} M **RhB** solution (red) and the solution (black) collected below the hydrogel.

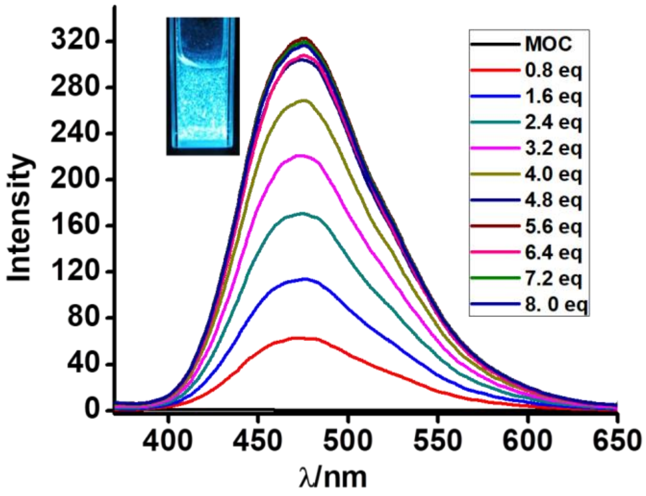

Supplementary Figure 46 | Interaction of MOC with DATPE in aqueous solution. The interaction of **MOC** and DATPE in solution is studied by gradually adding equivalents of DATPE into **MOC** (10 μM) solution. The **MOC** solution is non-emissive (black). After addition of 0.8 eq of DATPE into the aqueous solution of **MOC** a band at 474 nm (λ_{ex}= 350 nm) appears (red). With incremental addition of DATPE the emission intensity at 474 nm increases and saturates after addition of 6 eq DATPE. The solution becomes strong cyan emissive after titration.

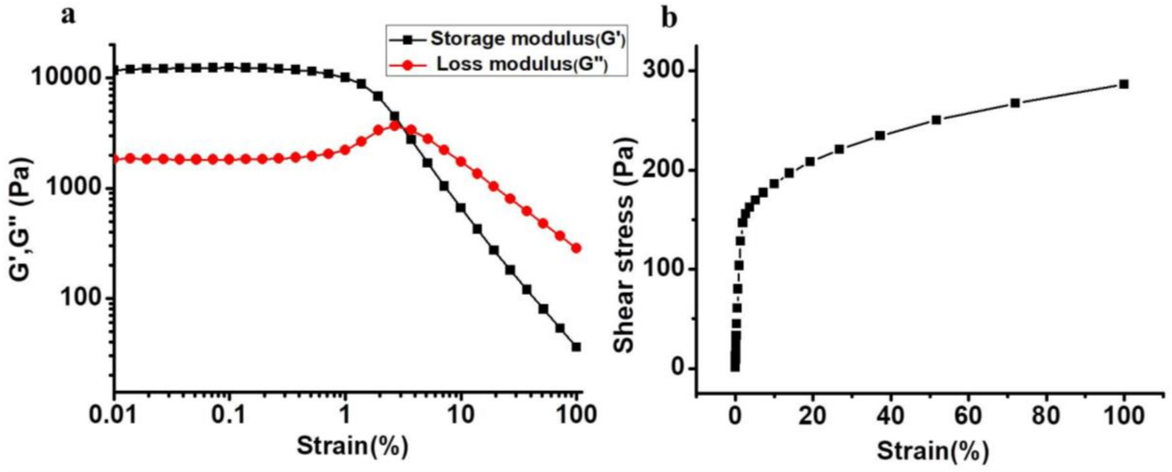

Supplementary Figure 47 | Rheology of MOC-G5 hydrogel. **a**, Oscillatory strain measurements (frequency=1.0 rads^{-1}) of **MOC-G5**, the squares (black) and circles (red) indicate storage (G') and loss modulus (G"), respectively. **b**, The stress *vs* stain plot of **MOC-G5**.

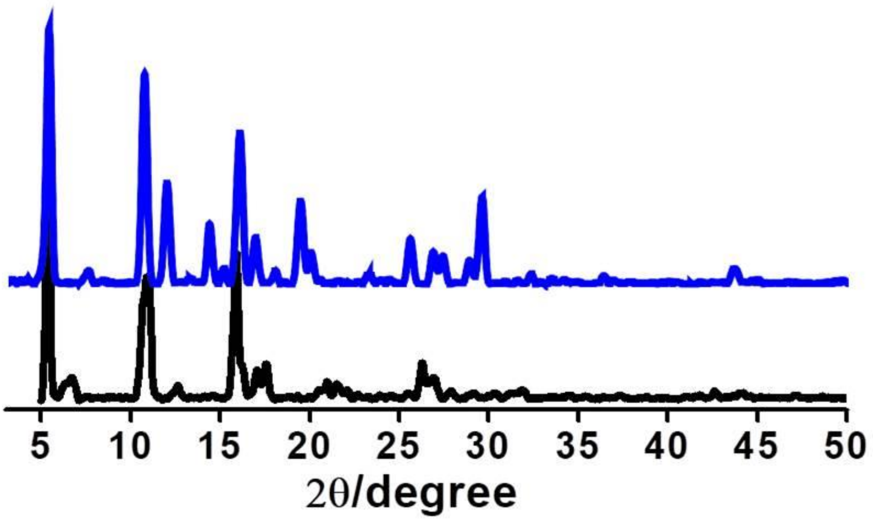

Supplementary Figure 48 | Comparison of PXRD pattern. PXRD pattern of **1** (black), **MOC-G5** xerogel (blue). Similarity in Bragg's reflections indicates stability of **MOC** in **MOC-G5** xerogel.

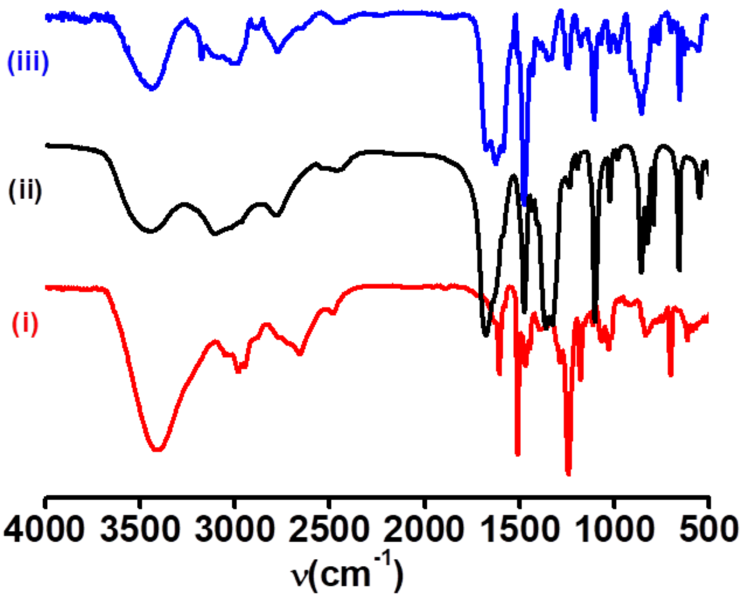

Supplementary Figure 49 | Comparision of FTIR spectra. FTIR spectra of DATPE (i), **1** (ii) and **MOC-G5** xerogel (iii).

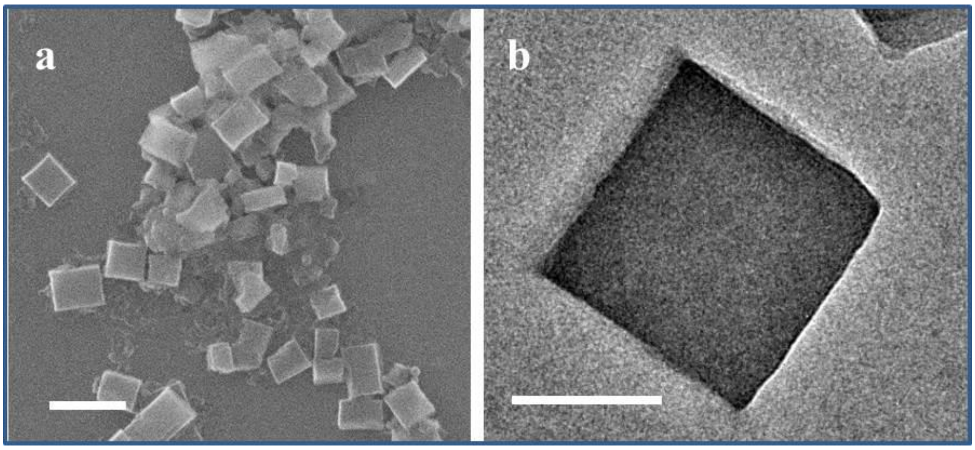

Supplementary Figure 50│Morphology of MOC-G5.a, FESEM image of **MOC-G5** xerogel showing presence of nanocubes over a large area. **b**, TEM image of **MOC-G5** xerogel showing the nanocube morphology.**a**, Scale bar = 1 μm and **b**, scale bar = 200 nm.

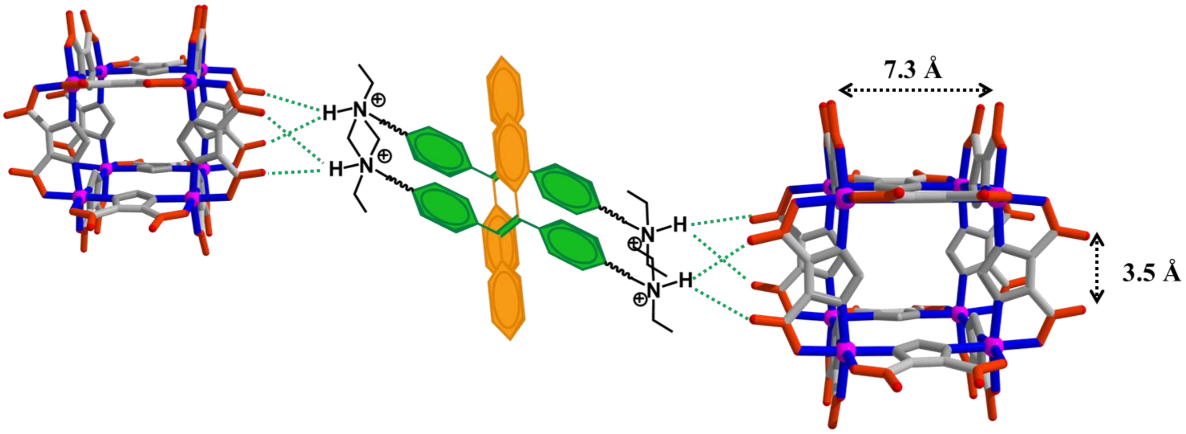

Supplementary Figure 51│ Charge-assisted H-bonding between MOC and DATPE. Schematic presentation showingpossible charge-assisted H-bonding interaction between DATPE and **MOCs**.

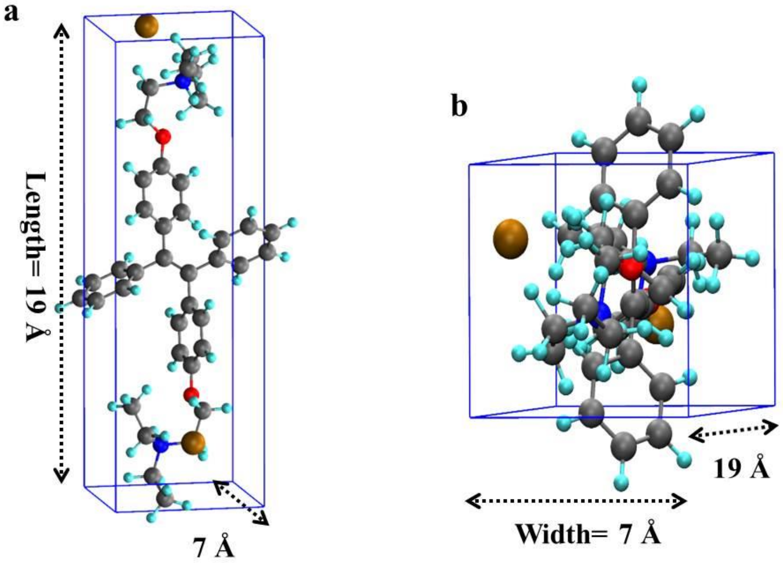

Supplementary Figure 52 │ Molecular dimensions of DATPE. a, The length of the DATPE molecule was calculated to be 19 Å. **b**, The width of the molecule was calculated to be 7 Å.

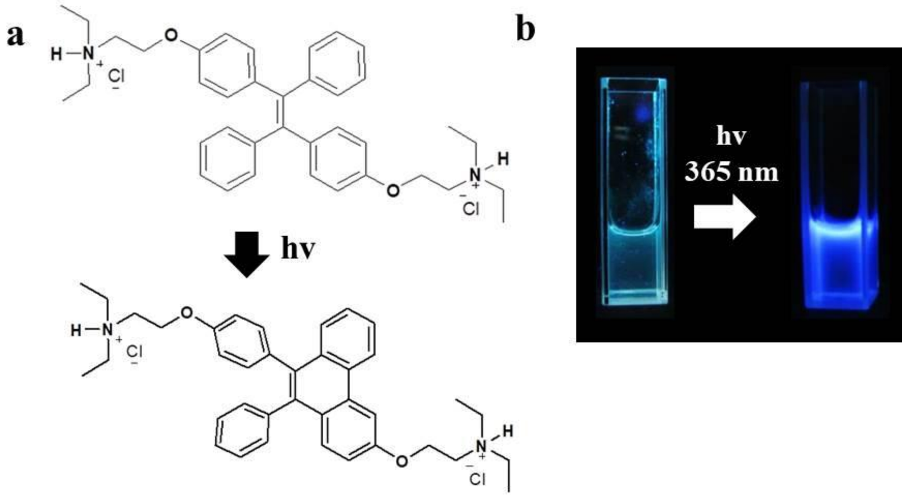

Supplementary Figure 53 │ Photo-cyclization of DATPE to DPPQA. a, Schematic showing the photo-cyclization of pure DATPE to DPPQA on photoirradiation. **b**, The aqueous solution of DATPE which is weekly-emissive, becomes intensely blue emissive after UV-light irradiation due to the formation of DPPQA.

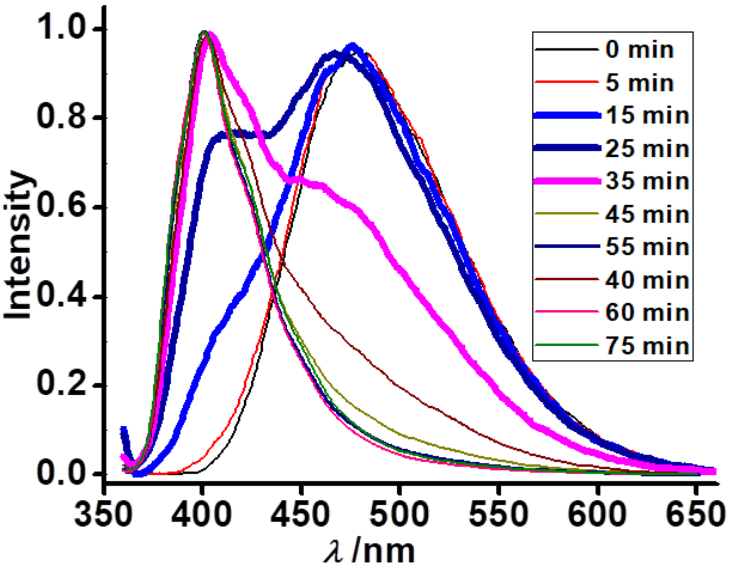

Supplementary Figure 54│ Change in emission spectra after photo-cyclization. Time dependant changes in emission spectra (normalized) of DATPE in water on irradiation with 365 nm light.

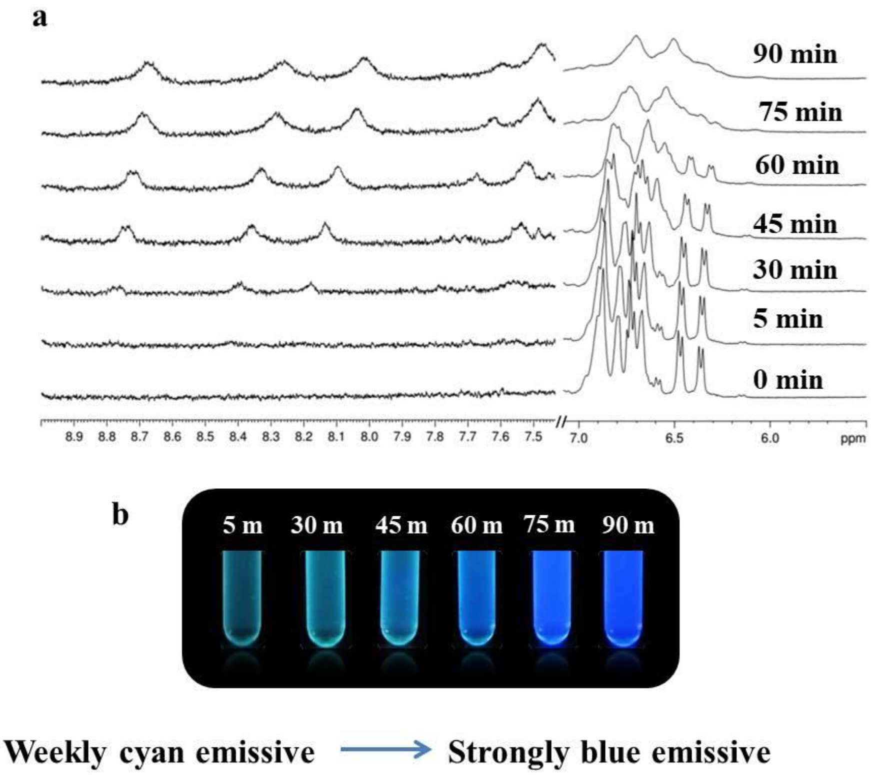

Supplementary Figure 55│ Monitoring photo-cyclization of DATPE with time. a, Changes in ^{1}H-NMR spectra of DATPE in D_2O at different intervals of photoirradiation. **b**, The corresponding images of DATPE in NMR tube under UV lamp.

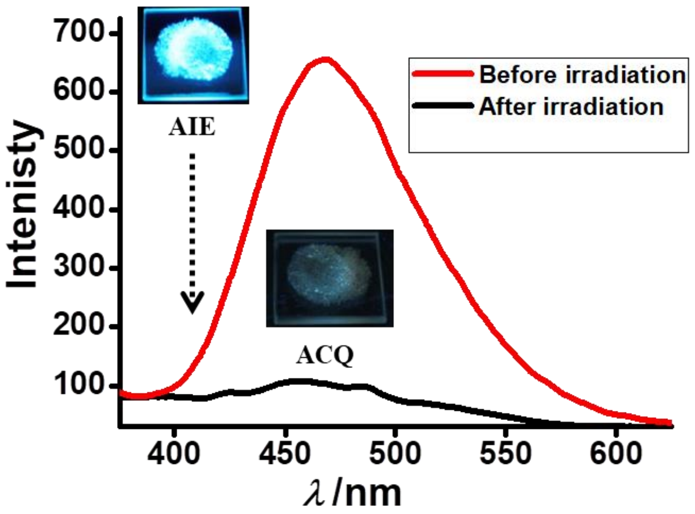

Supplementary Figure 56│ Change in emission of MOC-G5 after photoirradiation. Emission quenching of **MOC-G5** hydrogel coated on glass substrate on photoirradiation with 365 nm light source.

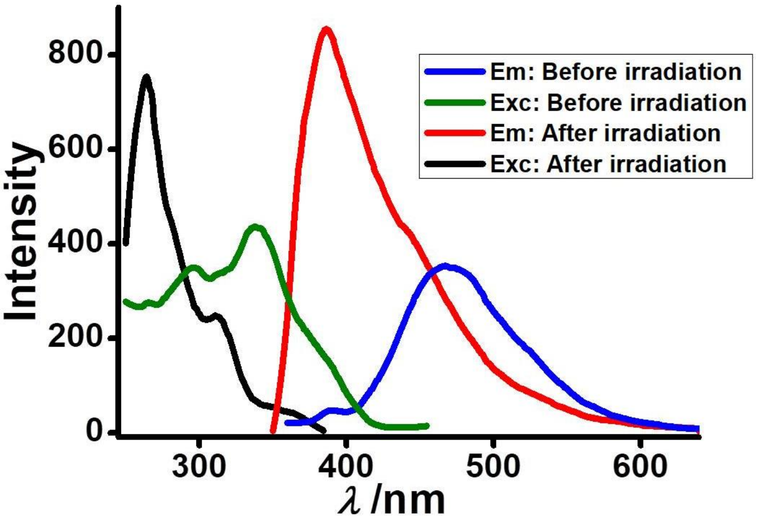

Supplementary Figure 57│ Emission and excitation spectra of MOC-G5 before and after photo-irradiation. Emission and excitation spectra of MOC-DATPE (blue, green) and MOC-DPPQA (red, black) hybrids dispersed in water before and after photoirradiation.

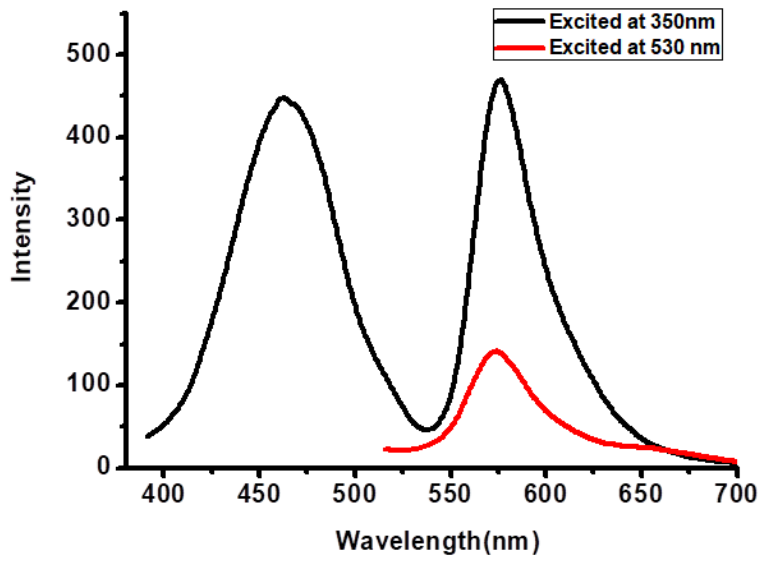

Supplementary Figure 58│ Energy transfer in mixed hybrid hydrogel. Emission spectra of **Rh6G$_{0.08\%}$@MOC-G5** hydrogel excited at 350 (black, indirect excitation) and 530 nm (red, direct excitation). Direct excitation of **Rh6G$_{0.08\%}$@MOC-G5** hydrogel shows less emission intensity compared to indirect excitation indicating energy transfer.

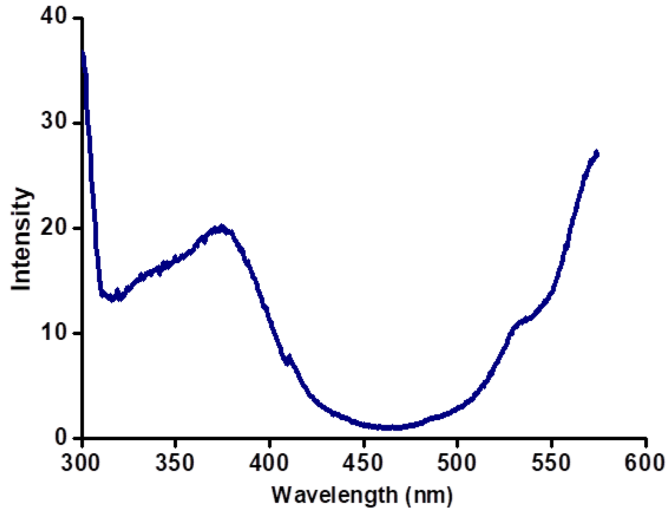

Supplementary Figure 59│Excitation spectra of Rh6G$_{0.08\%}$@MOC-G5. Excitation spectrum of **Rh6G$_{0.08\%}$@MOC-G5** collected at 580 nm, showing maximum intensity at 350 nm confirming the contribution of DATPE to the observed emission at 580 nm.

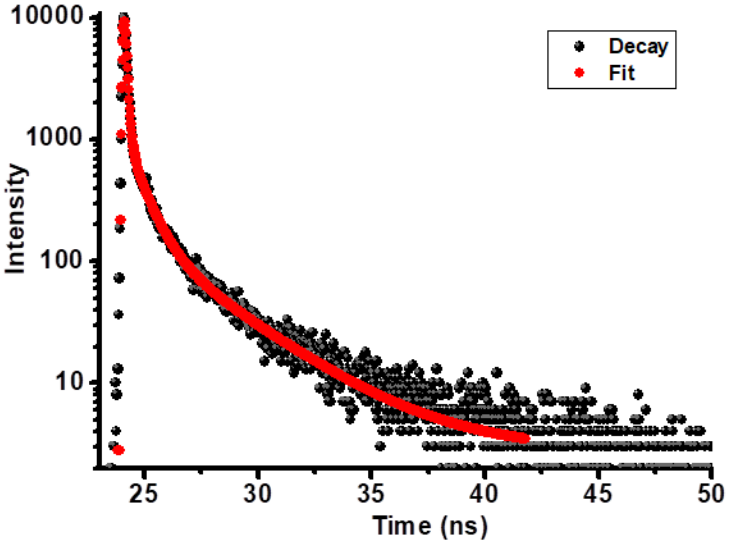

Supplementary Figure 60│Fluorescence decay profile of $Rh6G_{0.08\%}$@MOC-G5. Fluorescence decay profiles of **$Rh6G_{0.08\%}$@MOC-G5**(black) collected at 470 nm showing life time (τ) = 2.4 nm.

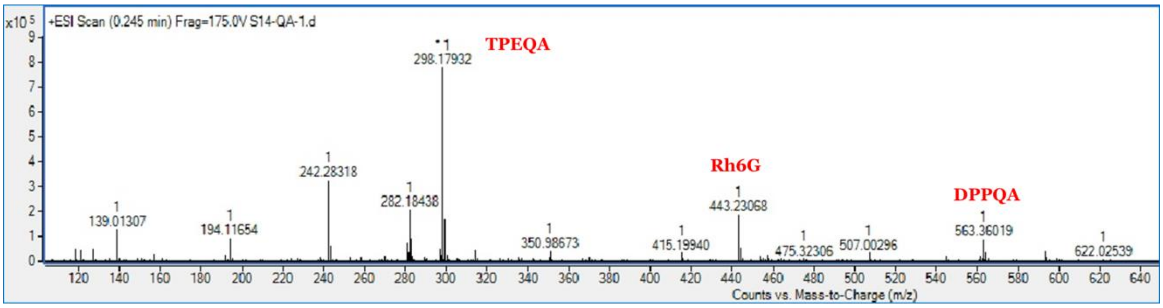

Supplementary Figure 61│ Formation of photo-cyclized product in $Rh6G_{0.08\%}$@MOC-G5. Positive ion acquisition mode HRMS of **$Rh6G_{0.08\%}$@MOC-G5** after 5 min of photo-irradiation. The hybridis dissolved in water. The HRMS shows presence of TPEQA, Rh6G and the photo-cyclized product(DPPQA).

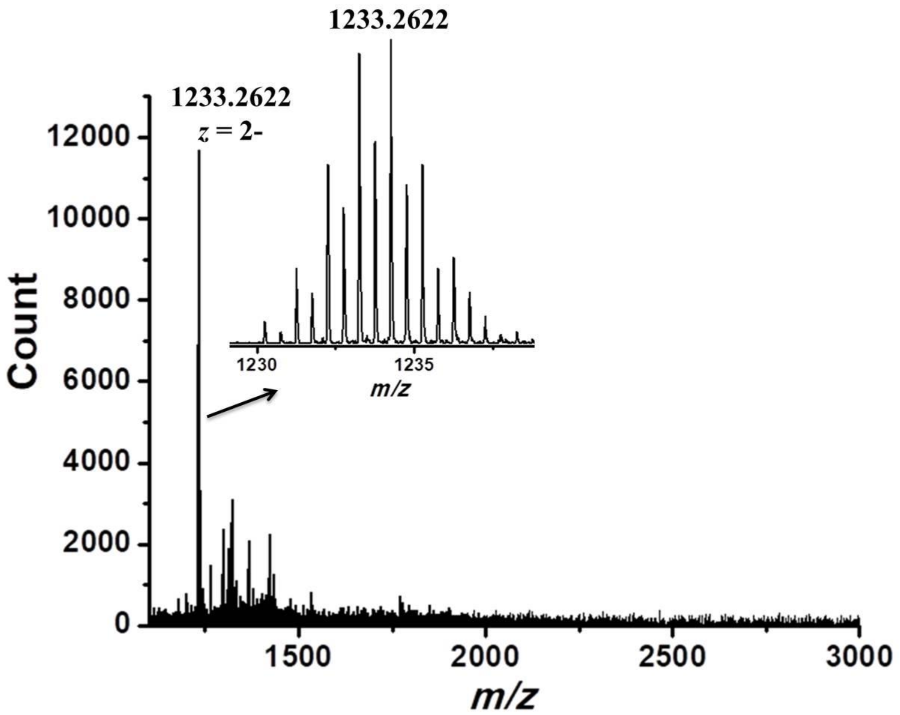

Supplementary Figure 62│Stability of MOC after photo-irradiation. The negative ion acquisition mode HRMS of photo-irradiated **MOC** showing peaks at *m/z*= 1233.2622 corresponding to $[(2Na^+)(8H^+)\{Ga_8(ImDC)_{12}\}(H_2O)]^{2-}$ moiety. This indicates stability of **MOC** after photo-irradiation.

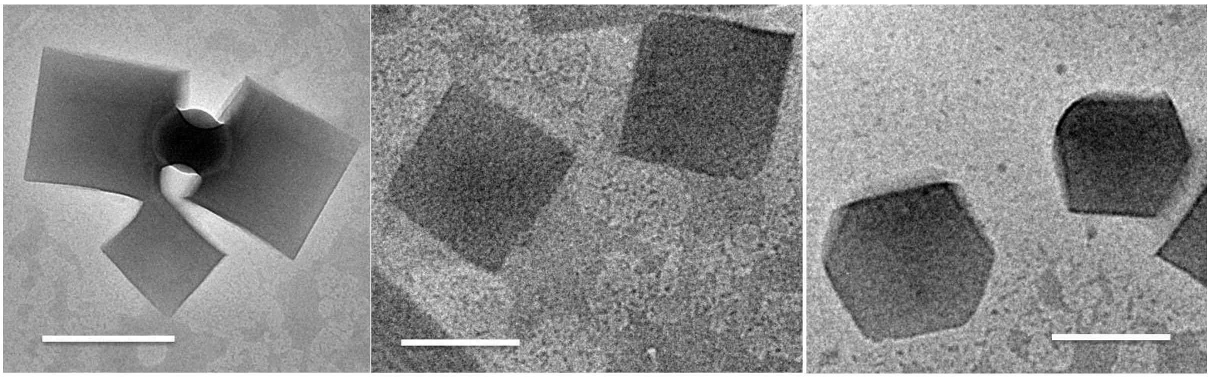

Supplementary Figure 63│Retention of nanocube morphology after photo-irradiation. TEM images of **Rh6G$_{0.08\%}$@MOC-G5** hydrogel after 5 min of photo-irradiation showing the nanocube morphology. **a**, Scale bar = 500 nm. **b** and **c**, Scale bar = 200 nm.

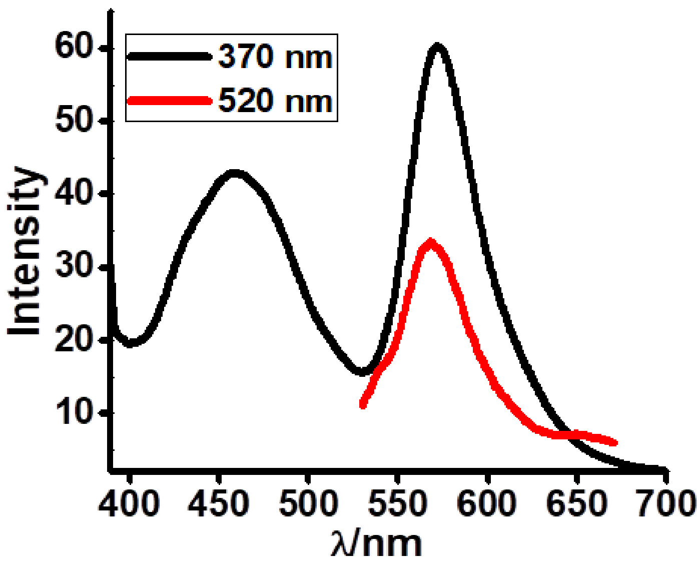

Supplementary Figure 64│ Energy transfer in white-light-emitting hydrogel. Emission spectra of white light emitting hydrogel (**Rh6G0.08%@MOC-G5** after 5 min of photo-irradiation) shows less emission intensity compared to indirect excitation indicating energy transfer.Excited at 370 (black, indirect excitation) and 520 nm (red, direct excitation).

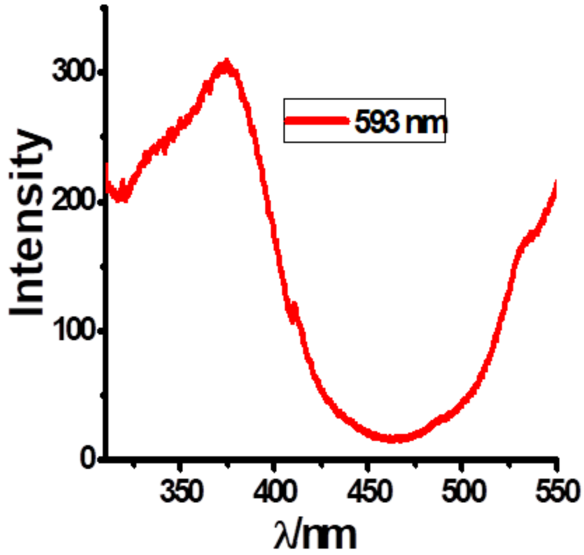

Supplementary Figure 65│Excitation spectrum of white-light-emitting hydrogel. Excitation spectrum of white light emitting gel collected at 593 nm.

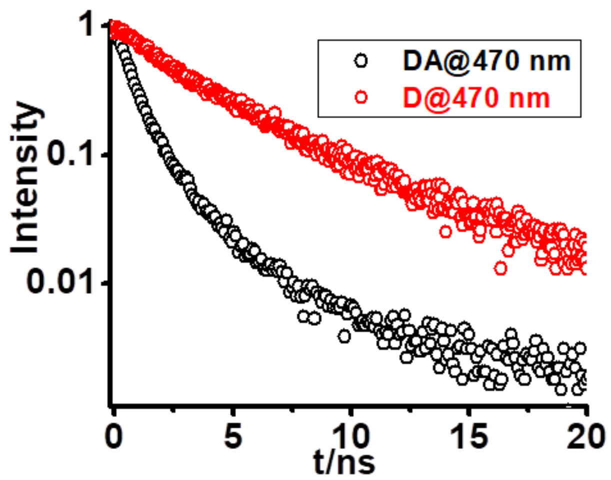

Supplementary Figure 66│ Fluorescence decay profiles. Fluorescence decay profiles of **MOC-G5** hydrogel (red) and white light emitting gel (black) collected at 470 nm.

Supplementary Discussion│ Calculation of fluorescence lifetime (τ), energy transfer efficiency (Φ_e) and energy transfer constants (k_e)

The weighted average fluorescence lifetimes were calculated using the following algorithm.

$\tau_{av} = (A_{w1}\tau_1 + A_{w2}\tau_2 + A_{w3}\tau_3)/(A_{w1} + A_{w2} + A_{w3})$, where $A_{wi} = A_i / \Sigma A_i$

τ and A are lifetime and amplitude, respectively, for multi-exponential fitting.

Fitting of fluorescence decays. Energy transfer efficiency, Φ_e.

$\Phi_e = k_e / (k_r + k_{nr} + k_e) = k_e / (k_o + k_e)$

where k_r, k_{nr}, and k_e = radiative decay, non-radiative decay, and energy transfer constants, respectively. The k_o and k_e values were found from the lifetimes of **MOC-G5** (collected at 474 nm, λ_{ex}= 370 nm) and **Rh6G$_{0.08\%}$@MOC-G5** (collected at 474 nm, λ_{ex}= 370 nm), respectively.

Life time of **MOC-G5** = τ_{direct} and lifetime of **Rh6G$_{0.08\%}$@MOC-G5** = $\tau_{indirect}$

$\tau_{direct} = 1/k_o$ and $\tau_{indirect} = 1/(k_o + k_e)$, respectively.

τ_{direct}=4.50 ns and $\tau_{indirect}$ = 2.44 ns.

Therefore, Φ_e = 45.8% and $k_e = 1.876\times10^8$ s^{-1}, for **Rh6G$_{0.08\%}$@MOC-G5** gel which is showing pink emission.

For white-light-emitting gel, τ_{direct}=4.50 ns and $\tau_{indirect}$ = 1.50 ns.

Therefore, Φ_e = 66.7% and $k_e = 4.446\times10^8$ s^{-1}.

Supplementary Reference

1. SMART (V 5.628), SAINT (V 6.45a), XPREP, SHELXTL; Bruker AXS Inc. Madison, Wisconsin, USA, 2004.

2.Sheldrick,G. M., Siemens Area Detector Absorption Correction Program, University of Göttingen, Göttingen, Germany(1994).

3.Altomare,A., Cascarano,G.,Giacovazzo,C., Gualaradi,A., *J. Appl.Cryst.*, **26**, 343 (1993).

4.Sheldrick,G. M., SHELXL-97, Program for Crystal Structure Solution and Refinement; University of Göttingen, Göttingen, Germany(1997).

5.Spek,A. L., *J. Appl.Cryst.*,**36**, 7(2003).

6.Farrugia,L. J., WinGX–A Windows Program for Crystal Structure Analysis, *J. Appl.Crystallogr.*,**32**, 837 (1999).
